# Compartment-specific distribution of human intestinal innate lymphoid cells is altered in HIV patients under effective therapy

**DOI:** 10.1371/journal.ppat.1006373

**Published:** 2017-05-15

**Authors:** Benjamin Krämer, Felix Goeser, Philipp Lutz, Andreas Glässner, Christoph Boesecke, Carolynne Schwarze-Zander, Dominik Kaczmarek, Hans Dieter Nischalke, Vittorio Branchi, Steffen Manekeller, Robert Hüneburg, Tobias van Bremen, Tobias Weismüller, Christian P. Strassburg, Jürgen K. Rockstroh, Ulrich Spengler, Jacob Nattermann

**Affiliations:** 1 Department of Internal Medicine I, University of Bonn, Bonn, Germany; 2 German Center for Infection Research (DZIF), Bonn, Germany; 3 Department of Surgery, University of Bonn, Bonn, Germany; 4 Department of Otolaryngology, University of Bonn, Bonn, Germany; Vaccine Research Center, UNITED STATES

## Abstract

Innate lymphocyte cells (ILCs), a novel family of innate immune cells are considered to function as key orchestrators of immune defences at mucosal surfaces and to be crucial for maintaining an intact intestinal barrier. Accordingly, first data suggest depletion of ILCs to be involved in human immunodeficiency virus (HIV)-associated damage of the intestinal mucosa and subsequent microbial translocation. However, although ILCs are preferentially localized at mucosal surfaces, only little is known regarding distribution and function of ILCs in the human gastrointestinal tract. Here, we show that in HIV(-) individuals composition and functional capacity of intestinal ILCs is compartment-specific with group 1 ILCs representing the major fraction in the upper gastrointestinal (GI) tract, whereas ILC3 are the predominant population in ileum and colon, respectively. In addition, we present first data indicating that local cytokine concentrations, especially that of IL-7, might modulate composition of gut ILCs. Distribution of intestinal ILCs was significantly altered in HIV patients, who displayed decreased frequency of total ILCs in ileum and colon owing to reduced numbers of both CD127(+)ILC1 and ILC3. Of note, frequency of colonic ILC3 was inversely correlated with serum levels of I-FABP and sCD14, surrogate markers for loss of gut barrier integrity and microbial translocation, respectively. Both expression of the IL-7 receptor CD127 on ILCs as well as mucosal IL-7 mRNA levels were decreased in HIV(+) patients, especially in those parts of the GI tract with reduced ILC frequencies, suggesting that impaired IL-7 responses of ILCs might contribute to incomplete reconstitution of ILCs under effective anti-retroviral therapy. This is the first report comparing distribution and function of ILCs along the intestinal mucosa of the entire human gastrointestinal tract in HIV(+) and HIV(-) individuals.

## Author summary

Current treatment regimens are very effective in blocking replication of the human immunodeficiency virus (HIV). However, due to ongoing activation of the immune system this often does not fully restore human health. The mechanisms underlying such activation of the immune system are only incompletely understood but are likely to include HIV-induced injury of the gut epithelial barrier and subsequent intrusion of gut flora components into the systemic circulation (microbial translocation). This study looks at the distribution and function of innate lymphoid cells (ILCs) in the gut mucosa of HIV(-) and HIV(+) patients as these cells are important for maintaining an intact gut epithelium. We found that composition of the intestinal ILC pool differs between the specific segments of the digestive tract and is significantly altered in HIV(+) patients. Furthermore, our data suggest a link between dys-regulated intestinal ILCs and loss of intestinal barrier function in HIV infection, thereby allowing for microbial translocation and systemic immune activation. This finding is new and highlights the important role played by the gut immune system in HIV-induced illness.

## Introduction

The gastrointestinal (GI) tract is the largest internal extension of the body’s surface and as such is constantly exposed to a myriad of environmental stimuli that include, but are not limited to microbes, dietary products and diverse inorganic materials [1]. Thus an important task of the GI tract is to segregate the underlying tissues from potentially harmful components of the environment. Accordingly, HIV-induced damage of the gut epithelial barrier resulting in increased microbial translocation [2,3] has been proposed to represent a major cause of HIV-induced systemic inflammation [4].

On the other hand, uptake of fluids and nutrients is vital, and thus some degree of permeability is essential. To ensure optimal transport of nutrients and fluids, while preventing translocation of commensals and pathogenic microorganisms the intestinal tract is also equipped with a highly complex, intrinsic immune system.

In this context, innate lymphocyte cells (ILCs), a novel family of innate immune cells, are of special interest as these cells are considered to function as key orchestrators of immune defences at mucosal surfaces and to be crucial for maintaining an intact intestinal barrier [5–7].

Based on the expression of specific transcription factors, cell-surface markers, and signature cytokines ILCs can be divided into 3 groups. IFN-γ producing, T-bet depending group 1 ILC (ILC1) encompass conventional natural killer (cNK) cells as well as CD127(+)ILC1, and the recently characterized CD103(+)ILC1 subset [8,9]. Group 2 ILCs (ILC2s) are a population of cells that preferentially produce type 2 cytokines, including IL-5 and IL-13, and require GATA3 [10]. Group 3 ILCs (ILC3s) can produce IL-17 and/or IL-22, and are dependent on RORγt [11].

In line with data obtained in mouse models [12,13] Simoni and co-workers recently demonstrated that phenotype and frequency of human ILC subsets differs between various tissues [14]. Moreover, there is increasing data suggesting that localization of ILC subsets in specific compartments relates to their roles in immune and inflammatory responses [13]. However, none of these studies performed a detailed analysis of ILC subsets in the different segments of the digestive tract and, thus, little is known regarding the complexity of human intestinal ILC subsets.

With respect to HIV infection there are first data demonstrating significant alterations of the ILC pool both in animal models as well as in human patients [15–20]. In simian immunodeficiency virus (SIV) infection a persistent loss of IL-17–producing ILCs as well as rapid decrease in NKp44+ ILC3 in the intestinal mucosa has been reported [15,16]. Similar findings have been made in humanized mice infected with HIV, which also displayed an HIV-induced reduction of ILC3 in the colon mucosa [17].

In patients with acute HIV infection Kløverpris et al. recently demonstrated a severe depletion of circulating ILCs, which only partly recovered after peak viremia. Of note, these authors found that increase in levels of intestinal fatty acid binding protein (I-FABP), a marker for dys-integrity of the intestinal epithelial barrier, coincides with loss of circulating ILCs, suggesting a mechanistic link between depletion of ILCs and epithelial gut breakdown in HIV infection [18]. However, little data were provided regarding intestinal ILCs in HIV patients.

Here, we studied ILCs over the entire length of the gastrointestinal tract and show that composition and function of the intestinal ILC pool in HIV(-) individuals is compartment-specific. Moreover, we demonstrate that HIV patients under effective therapy display a dys-regulated distribution of intestinal ILCs, which might be involved in loss of intestinal barrier integrity.

## Results

### Compartment-specific distribution and function of intestinal ILCs in HIV(-) individuals

**Fig 1:**
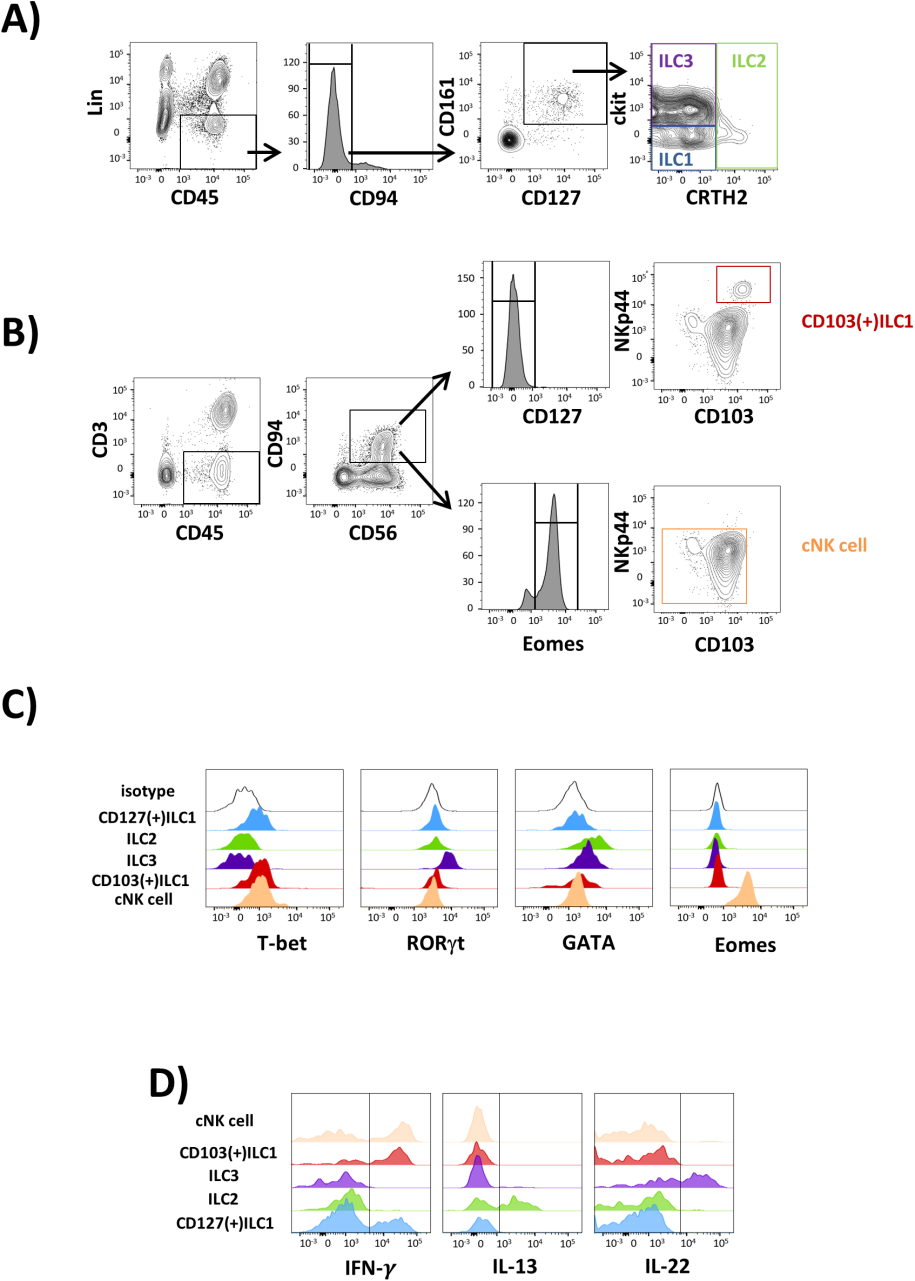
Characterisation of human ILC subsets in intestinal biopsy specimen. **(A)** Representative FACS plots showing the gating strategy from singlet lymphocytes to the ILC1 (blue), ILC2 (green), and ILC3 (violet) populations. **(B)** Gating strategy used for the identification of CD103(+)ILC1 (red) and conventional NK (cNK) cells (orange). **(C)** Expression of the transcription factors T-bet, RORγt, GATA, and Eomes within intestinal CD127(+)ILC1 (blue), CD103(+)ILC1 (red), ILC2 (green), ILC3 (violet), and cNK cells (orange). **(D)** Production of IFN-γ, IL-13, and IL-22, respectively, in PMA/ ionomycin activated intestinal CD127(+)ILC1 (blue), CD103(+)ILC1 (red), ILC2 (green), ILC3 (violet), and cNK cells (orange). Due to low frequencies pooled samples of three donors had to be studied in the case of ILC2.

First, we tested whether the currently defined ILC subsets can be analysed in biopsy specimen by flow cytometry. Following standard gating protocols [11,21] we identified five phenotypically different ILC populations:

lineage(Lin)(-)CD45(+)CD127(+)CD161(+)CRTH2(-)c-kit(-) cells representing CD127(+)ILC1, Lin(-)CD45(+)CD127(+)CD161(+)CRTH2(+)c-kit(-/+) ILC2, Lin(-)CD45(+)CD127(+)CD161(+)CRTH2(-)c-kit(+) ILC3 (Fig 1A), CD3(-)CD45(+)CD56(+)CD94(+)CD127(-)CD103(+)NKp44(+) intra-epithelial ILC1 (CD103(+)ILC1) as well as CD3(-)CD45(+)CD56(+)CD94(+) NKp44(-) conventional natural killer (cNK) cells (Fig 1B and S1 Fig). To further confirm the identity of these lymphocyte populations expression of transcription factors was studied (Fig 1C). Here, we observed CD127(+)ILC1 and CD103(+)ILC1 to express T-bet but to be negative for RORγT, Eomes, and GATA. ILC2 were found to be GATA(+)T-bet(-)RORγt(-)Eomes(-), whereas ILC3 displayed an T-bet(-) RORγt(+)Eomes(-) phenotype. In addition, we tested cytokine production of PMA/ionomycin-stimulated ILCs (Fig 1D). As could be expected we found ILC1 subsets to display a robust IFN-γ production, similar to that observed in conventional NK cells, whereas ILC2 and ILC3 were characterised by production of IL-13 and IL-22, respectively. Taken together, these data confirmed a precise identification of human ILC subsets isolated from intestinal biopsies.

**Fig 2:**
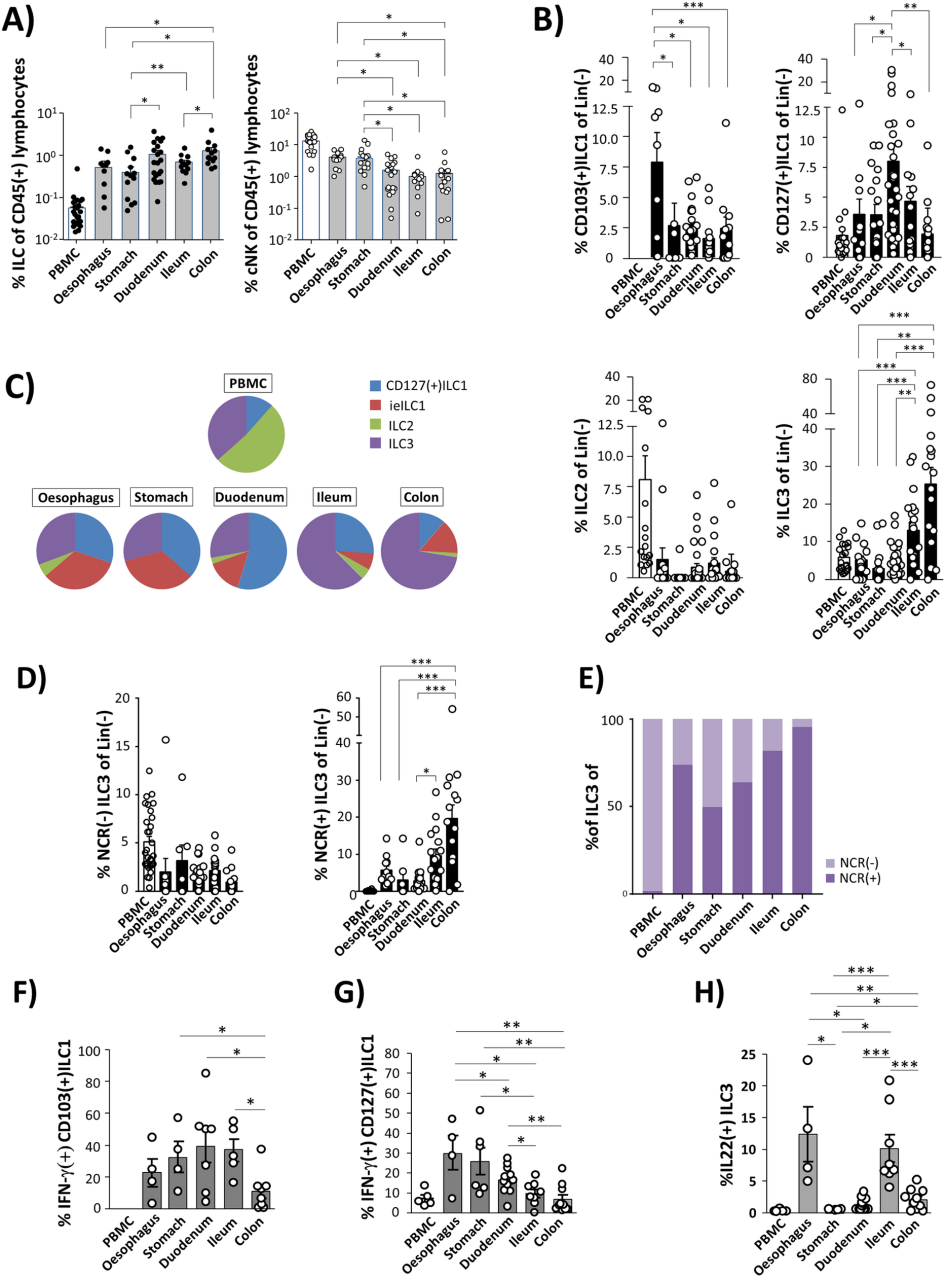
Compartment-specific distribution and function of intestinal ILCs in HIV(-) individuals. **(A)** Frequency of ILCs (black dots, left panel) and cNK cells (white dots; right panel) expressed as percentage of CD45(+) lymphocytes in the different parts of the human GI tract (ILCs: oesophagus, n = 10; stomach n = 15; duodenum, n = 21; ileum, n = 13; colon: n = 13; cNK: oesophagus, n = 12; stomach n = 15; duodenum, n = 21; ileum, n = 14; colon: n = 13; grey columns) in comparison to peripheral blood (n = 16; white columns). **(B)** Distribution of CD103(+)ILC1 (upper left panel), CD127(+)ILC1 (upper right panel), ILC2 (lower left panel), and ILC3 (lower right panel) in the human intestinal tract (black columns) and the circulating blood (white columns). **(C)** Composition of CD127(+)ILC1 (blue), intraepithelial ILC1 (red), ILC2 (green) and ILC3 (violet) in the peripheral blood and the different segments of the gastrointestinal tract. **(D)** Distribution of NCR(-) (left panel) and NCR(+) ILC3 (right panel) in the peripheral blood and the different segments of the gastrointestinal tract. **(E)** Percentage comparison between NCR(-) and NCR(+) ILC3 subsets in those compartments. For functional characterisation ILCs derived from PBMC or biopsy samples (oesophagus, stomach, duodenum, Ileum and colon) obtained from HIV(-) individuals, were stimulated with PMA/ionomycin and then tested for IFN-γ production in CD103(+)ILC1 **(F)**, CD127(+)ILC1 **(G)**,or IL-22 production in ILC3 **(H)**, respectively. * p≤0.05; ** p≤0.01; *** p≤0.001 (FDR-adjusted P values, parametric ANOVA).

Using this approach, we next studied ILC distribution in the different compartments of the GI tract in HIV(-) individuals. For clarity reasons, in the following the term “total ILCs” will be used to define the sum of all ILC subsets except cNK cells, which will be described separately. In contrast to cNK cells, the frequency of total ILCs increased from the oral to the distal parts of the GI tract and was highest in the ileum and colon (Fig 2A). Analysing the specific ILC subsets separately (Fig 2B and S2 Fig), we found frequency of CD103(+)ILC1 to be highest in the oesophagus and to decrease towards the terminal ileum, whereas CD103(+)ILC1 frequencies in the left-sided colon were similar to that observed in the stomach. CD127(+)ILC1 frequencies did not differ significantly between the analysed compartments with the exception of duodenal tissues, which contained the highest proportion of CD127(+)ILC1. ILC2 represented a minor ILC fraction in all studied parts of the alimentary tract. This was also confirmed when *lamina propria* and intra-epithelial ILCs were studied separately in duodenal resectates obtained from patients that underwent tumor surgery (S3A Fig). Moreover, we could exclude that the protocol used for isolating ILCs from biopsy specimen might affect detection of group 2 ILCs (S3 Fig). With respect to frequencies of ILC3, we observed a steady increase of from the upper part of the GI tract towards the colon, where the highest ILC3 numbers were observed. These differences were mainly attributable to the NCR(+) subset, whereas frequencies of NCR(-)ILC3 did not differ significantly between the different segments of the GI tract (Fig 2D and 2E). In summary, these data demonstrate a compartment-specific composition of the intestinal ILC pool with group 1 ILCs representing the major fraction in the upper parts of the human GI tract, whereas ILC3 are the predominant population in ileum and colon, respectively (Fig 2C).

Next, we compared the functional capacity of ILC subsets in the different compartments of the gut by analysing cytokine production of PMA/ionomycin-stimulated cells. As is shown in Fig 2F, we observed CD103(+)ILC1 in the stomach, the duodenum, and the ileum to display high IFN-γ production, whereas colon CD103(+)ILC1 were found to have a rather low capacity to produce IFN-γ. Similar findings were made regarding frequency of IFN-γ-expressing CD127(+)ILC1, which was found to decrease steadily from the oesophagus towards the left-sided colon (Fig 2G). A more detailed analysis revealed that this effect was mainly attributable to the NCR(-)CD127(+)ILC1 subset (S4 Fig). With respect to ILC3 we observed production of IL-22 to be high in cells isolated from the oesophagus and ileum mucosa, respectively, but low in stomach and duodenal ILC3, whereas colon ILC3 displayed an intermediate IL-22 production following stimulation with PMA/ionomycin (Fig 2H). This segment-specific functional capacity of ILC3 was especially seen in the NCR(+) subset (S4C Fig). Frequency of ILC2 was too low to enable reliable functional analysis.

### IL-7 / IL-15 and composition of the of intestinal ILC pool

**Fig 3:**
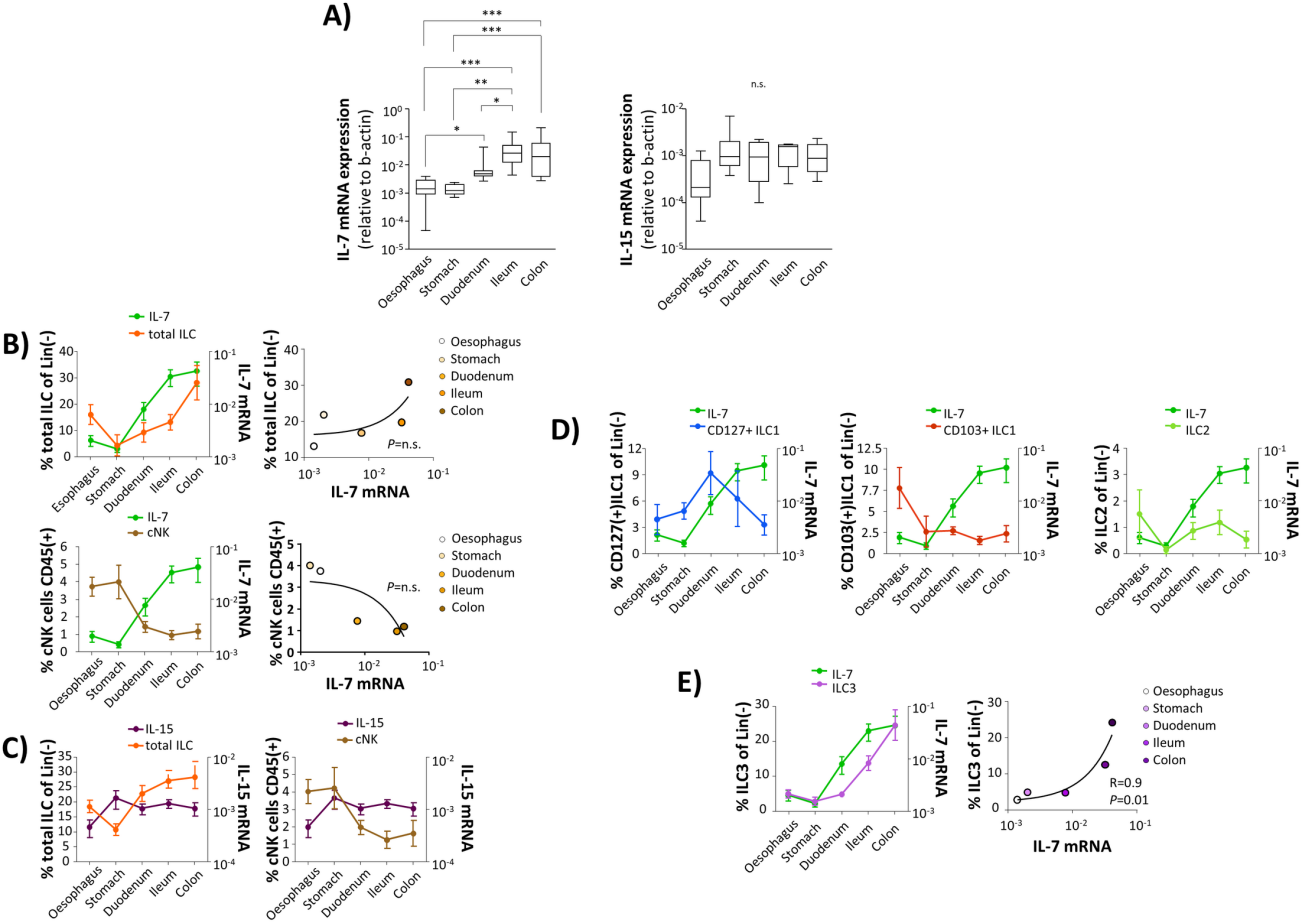
Local IL-7 levels and composition of the intestinal ILC pool in HIV(-) individuals. **(A)** Mucosal mRNA expression levels of IL-7 (left panel) and IL-15 (right panel) were analysed in the oesophagus (n = 7), the stomach (n = 9), the duodenum (n = 14), the ileum (n = 12), and the colon (n = 10), respectively (* p≤0.05; ** p≤0.01; *** p≤0.001 (FDR-adjusted P values, parametric ANOVA). Cytokine mRNA expression levels are expressed in relation to expression levels of beta-actin. **(B)** Compartment-specific IL-7mRNA levels (green) in comparison to frequency of total ILCs (orange; left panels; oesophagus (n = 6), stomach (n = 7), duodenum (n = 10), ileum (n = 9), and colon (n = 8)) and cNK cells (brown; right panels; oesophagus (n = 6), stomach (n = 7), duodenum (n = 9), ileum (n = 9), and colon (n = 7)) in the different segments of the GI tract. The right panels depict correlation between local IL-7 mRNA levels and ILC (upper panel) or cNK cells frequency (lower panel), respectively. Pearson correlation coefficient was computed for analysing correlations (right panels).**(C)** Relation between mucosal IL-15 mRNA expression levels (violet) and frequency of total ILCs (orange; left panels; oesophagus (n = 6), stomach (n = 7), duodenum (n = 10), ileum (n = 9), and colon (n = 8)) and cNK cells (brown; right panels; oesophagus (n = 6), stomach (n = 7), duodenum (n = 9), ileum (n = 9), and colon (n = 7)) in the different segments of the GI tract. **(D)** Local IL-7 mRNA concentrations (green) in relation to frequency of CD127(+)ILC1 (blue; upper left panel), CD103(+)ILC1 (coppery; upper right panel), ILC2 (lime green; lower left panel), and ILC3 (violet; lower right panel).; oesophagus (n = 6), stomach (n = 7), duodenum (n = 10), ileum (n = 9), and colon (n = 8). Pearson correlation coefficient was computed for analysing correlations. **(E)** Compartment-specific IL-7mRNA levels (green) in comparison to frequency of ILC3 (violet; left panel; oesophagus (n = 6), stomach (n = 7), duodenum (n = 10), ileum (n = 9), and colon (n = 8) in the different segments of the GI tract. The right panel depicts correlation between local IL-7 mRNA levels and ILC3 frequency. Pearson correlation coefficient was computed for analysing correlations.

IL-7 has been shown to be critical for the development of ILC2 and ILC3 subsets, whereas IL-15 plays an important role in the development of cNK cells and CD127(+)ILC1 [22,23]. Thus, we tested the hypothesis that local differences in cytokine concentrations might be involved in fine-tuning of the compartment-specific ILC homeostasis. Indeed, we observed mucosal IL-7 mRNA expression to differ significantly between the specific segments of the GI tract (Fig 3 and S5 Fig) with increasing concentrations from the proximal to the distal parts (Fig 3A, left panel), whereas no such differences were found for IL-15 (Fig 3A, right panel). More importantly, this increase in IL-7 mRNA expression levels from the proximal to the distal parts of the GI tract paralleled the increase in frequency of total ILCs (Fig 3B, upper left panel) but was negatively associated with frequency of intestinal cNK cells (Fig 3B, lower left panel) although none of these associations reached statistical significance. No associations were found for IL-15 (Fig 3C).

**Fig 4:**
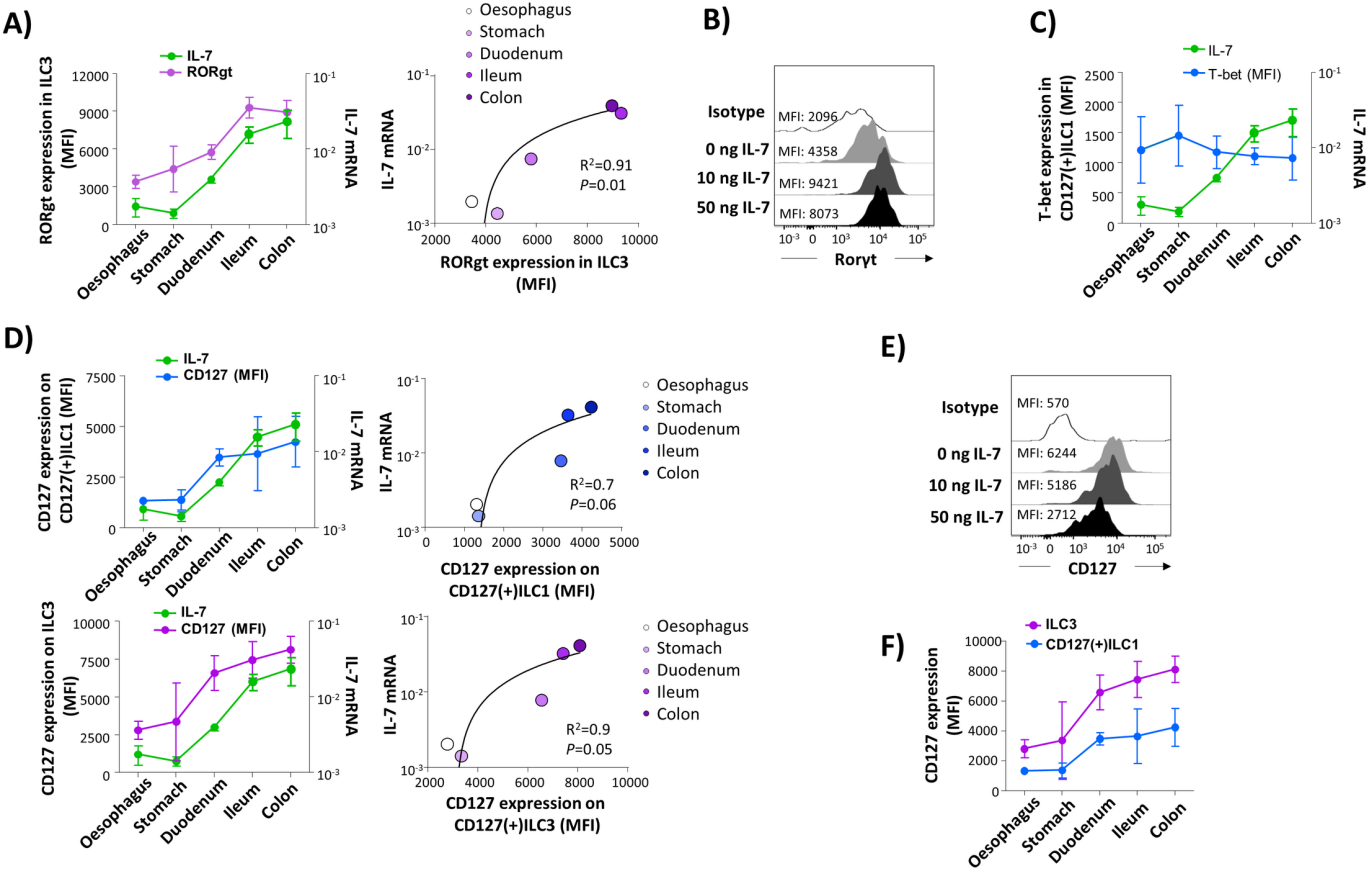
Local IL-7 levels and segment-specific characteristics of intestinal ILCs in HIV(-) individuals. **(A)** Relation between local IL-7 mRNA concentrations (green) and expression of RORγt in ILC3 (violet). Expression of RORγt is given as mean fluorescence intensity (MFI).oesophagus (n = 5), stomach (n = 7), duodenum (n = 6), ileum (n = 7), and colon (n = 6). **(B)** *In vitro* effect of increasing concentrations of recombinant IL-7 (0, 10, 50 ng/ml) expression on RORγt expression in tonsil ILC3. **(C)** Relation between local IL-7 mRNA concentrations (green) and expression of T-bet (MFI; blue) within intestinal CD127(+)ILC1. oesophagus (n = 5), stomach (n = 6), duodenum (n = 7), ileum (n = 7), and colon (n = 6)**(D)** Association between CD127 surface expression (MFI) on CD127(+)ILC1 (left panel; blue) and ILC3 (right panel; violet), respectively, and IL-7 mRNA concentrations (green). oesophagus (n = 6), stomach (n = 7), duodenum (n = 10), ileum (n = 9), and colon (n = 8) **(E)** *In vitro* effect of increasing concentrations of recombinant IL-7 (0, 10, 50 ng/ml) on surface expression of CD127. **(F)** CD127 surface expression (MFI) on ILC3 (violet;) compared to that of CD127(+)ILC1 (blue). oesophagus (n = 6), stomach (n = 7), duodenum (n = 10), ileum (n = 9), and colon (n = 8). Pearson correlation coefficient was computed for analysing correlations.

When ILC subsets were studied separately, we found that only frequency of ILC3 displayed a significant association with local concentrations of IL-7 mRNA (Fig 3E and S5C Fig). A direct link between intestinal IL-7 levels and ILC3s was further supported by our finding that within ILC3 expression of the transcription factor RORγt, which has been shown to be essential for ILC3 differentiation [24,25], was significantly correlated with IL-7 levels (Fig 4A). Moreover, we found *in vitro* stimulation of ILC3 with recombinant IL-7 to result in an increased RORγt expression (Fig 4B), resembling findings presented by Younas et al. who demonstrated IL-7 to increase RORγt mRNA levels in human regulatory T cells [26]. No such association could be found with respect to T-bet expression (Fig 4C).

Regarding expression of the IL-7 receptor CD127 we observed a positive correlation with IL-7 mRNA levels in ILC3 (Fig 4D, lower panels). This was a somewhat counterintuitive finding since IL-7 has been shown to induce receptor internalization in CD8(+) T cells [27]. Thus, we next tested whether IL-7 mediated regulation of CD127 expression in ILCs might differ from that in CD8(+) T cells. However, *in vitro* stimulation of ILCs with IL-7 was found to reduce surface expression of CD127 in a dose-dependent fashion, suggesting additional factors to be involved in compartment-specific modulation of CD127 expression on intestinal ILCs (Fig 4E). Accordingly, a similar association between CD127 expression and intestinal IL-7 mRNA levels was seen in CD127(+)ILC1 (Fig 4D, upper panels), although frequency of this ILC subset did not correlate with mucosal IL-7 concentrations. However, surface expression of CD127 was lower on ILC1 than on ILC3 (Fig 4F), which might reflect T-bet-mediated suppression of CD127 expression as has been shown in Tbx21^−/−^Rag2^−/−^ ulcerative colitis mice [28].

Taken together, these data suggest that local IL-7 concentrations might modulate composition of the intestinal ILC pool. However, given the complexity of the intestinal cytokine compartment [29] it is likely that cytokines other than IL-7 also may play a role in this context, although IL-7 displayed the strongest association with intestinal ILCs in our analysis (S6 Fig).

### Compartment-specific dys-regulation of the intestinal ILC pool in HIV infection

**Fig 5:**
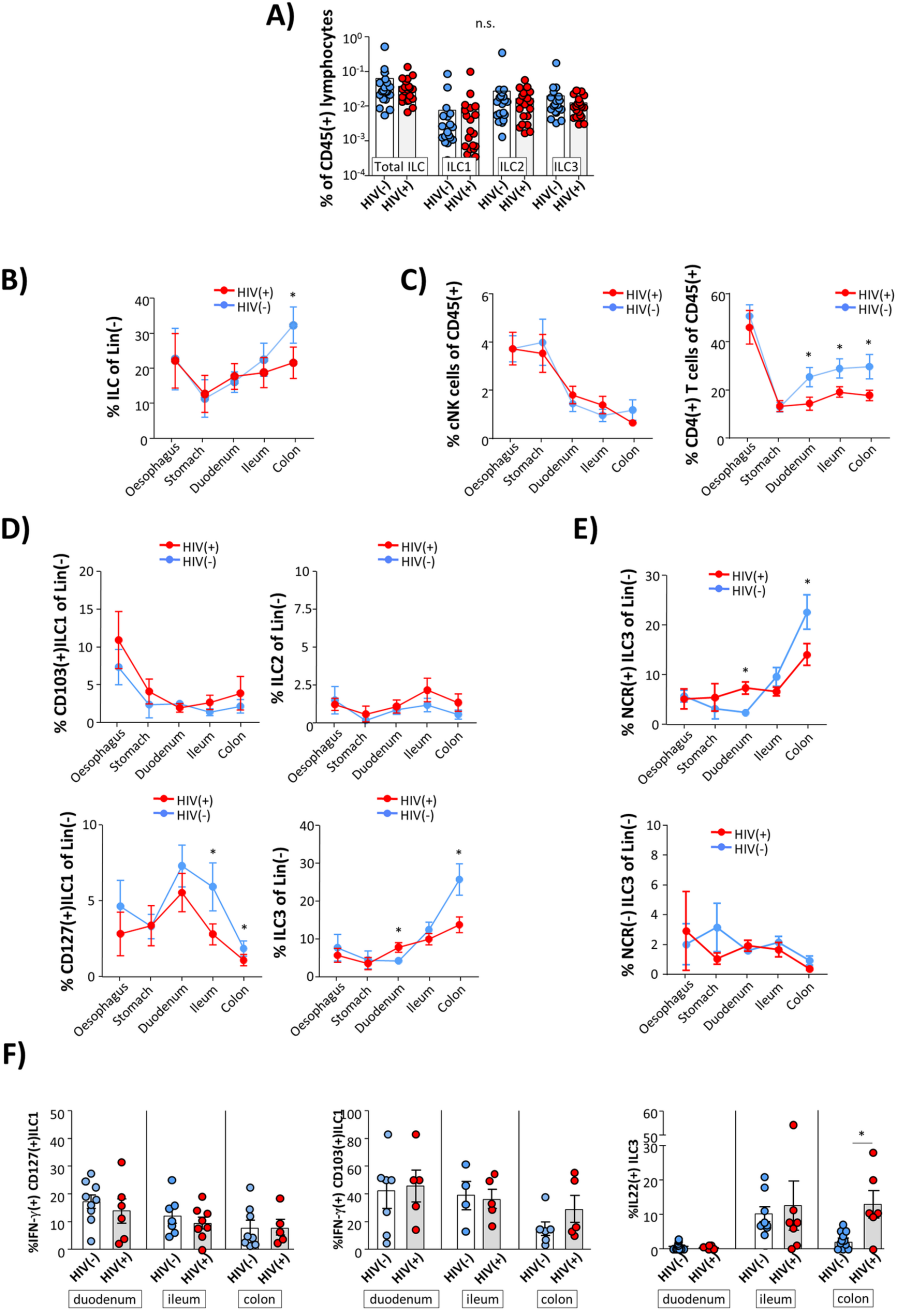
Alterations of the intestinal ILC pool in HIV(+) individuals. **(A)** Frequency of ILCs in the peripheral blood of HIV(-) controls (blue; n = 19) in comparison to HIV patients (red; n = 20) under effective cART (ns, not significant). **(B)** Frequency of total ILCs in the different segments of the GI tract in HIV(-) controls compared to HIV patients (* p≤0.05). oesophagus: HIV(-) n = 12, HIV(+) n = 9; stomach HIV(-) n = 15, HIV(+) n = 15; duodenum: HIV(-) n = 21, HIV(+) n = 19; ileum: HIV(-) n = 15, HIV(+) n = 16; colon: HIV(-) n = 13, HIV(+) n = 17. **(C)** Frequency of cNK cells (left panel) and CD4(+) T cells (right panel) in the different segments of the GI tract in HIV(-) controls compared to HIV patients. cNK cells: oesophagus: HIV(-) n = 12, HIV(+) n = 10; stomach HIV(-) n = 15, HIV(+) n = 15; duodenum: HIV(-) n = 21, HIV(+) n = 19; ileum: HIV(-) n = 13, HIV(+) n = 16; colon: HIV(-) n = 13, HIV(+) n = 17. CD4(+) T cells: HIV(-) n = 9, HIV(+) n = 9; stomach HIV(-) n = 12, HIV(+) n = 15; duodenum: HIV(-) n = 18, HIV(+) n = 18; ileum: HIV(-) n = 10, HIV(+) n = 17; colon: HIV(-) n = 11, HIV(+) n = 18. **(D)** Frequency of CD103(+)ILC1 (upper left panel), ILC2 (upper right panel), CD127(+)ILC1 (lower left panel), and ILC3 (lower right panel; in the different segments of the GI tract in HIV(-) controls compared to HIV patients. CD103(+)ILC1: oesophagus: HIV(-) n = 15, HIV(+) n = 10; stomach HIV(-) n = 17, HIV(+) n = 16; duodenum: HIV(-) n = 31, HIV(+) n = 22; ileum: HIV(-) n = 17, HIV(+) n = 15; colon: HIV(-) n = 18, HIV(+) n = 16. CD127(+)ILC1: oesophagus: HIV(-) n = 15, HIV(+) n = 10; stomach HIV(-) n = 16, HIV(+) n = 16; duodenum: HIV(-) n = 31, HIV(+) n = 22; ileum: HIV(-) n = 17, HIV(+) n = 15; colon: HIV(-) n = 18, HIV(+) n = 16. ILC2: oesophagus: HIV(-) n = 15, HIV(+) n = 10; stomach HIV(-) n = 16, HIV(+) n = 16; duodenum: HIV(-) n = 31, HIV(+) n = 22; ileum: HIV(-) n = 17, HIV(+) n = 15; colon: HIV(-) n = 18, HIV(+) n = 16. ILC3: oesophagus: HIV(-) n = 15, HIV(+) n = 10; stomach HIV(-) n = 17, HIV(+) n = 16; duodenum: HIV(-) n = 30, HIV(+) n = 22; ileum: HIV(-) n = 19, HIV(+) n = 15; colon: HIV(-) n = 19, HIV(+) n = 16. **(E)** Frequency of NCR(+) (upper panel) and NCR(-) ILC3 (lower panel) in the different segments of the GI tract in HIV(-) controls compared to HIV patients. NCR(+) ILC3: oesophagus: HIV(-) n = 15, HIV(+) n = 10; stomach HIV(-) n = 17, HIV(+) n = 16; duodenum: HIV(-) n = 30, HIV(+) n = 22; ileum: HIV(-) n = 19, HIV(+) n = 15; colon: HIV(-) n = 19, HIV(+) n = 16. NCR(-) ILC3: oesophagus: HIV(-) n = 15, HIV(+) n = 10; stomach HIV(-) n = 17, HIV(+) n = 16; duodenum: HIV(-) n = 30, HIV(+) n = 22; ileum: HIV(-) n = 19, HIV(+) n = 15; colon: HIV(-) n = 19, HIV(+) n = 16. **(F)** Functional activity of PMA/ ionomycin-stimulated ILC subsets (left panel: IFN-γ(+)CD127(+)ILC1; middle panel: IFN-γ(+)CD103(+)ILC1; right panel: IL-22(+) ILC3) isolated from the duodenum, the ileum, and the colon, respectively in HIV(+) patients and HIV(-) individuals. * p≤0.05; ** p≤0.01; *** p≤0.001 (FDR-adjusted P values, parametric ANOVA).

With respect to circulating blood, we observed slightly decreased frequencies of ILC2 and ILC3 in HIV infection. However, these differences did not reach statistical significance (Fig 5A and S8 Fig). In contrast, we observed a significant dys-regulation of the intestinal ILC pool in HIV patients under cART compared to healthy controls. As is shown in Fig 5B and S7 Fig we found HIV infection to be associated with significantly decreased frequencies of total ILCs in the colon, whereas no such differences were observed in the proximal parts of the GI tract. This pattern of HIV-associated alterations was specific to the ILC pool as frequency of cNK cells was not significantly altered, whereas number of CD4(+) T cells was significantly reduced in duodenum, ileum, and colon of HIV(+) patients (Fig 5C). A more detailed analysis demonstrated that specifically frequencies of CD127(+)ILC1 (Fig 5D, lower left panel) and ILC3 (Fig 5D, lower right panel) were altered in HIV(+) individuals. Again, these HIV-associated alterations of the ILC3 pool were mainly attributable to the NCR(+) subset (Fig 5E, upper panel). In contrast to colon, we found ILC3 frequencies to be significantly increased in the duodenum of HIV patients. Of note, this increase was specific to the ILC3 subset as frequency of duodenal CD127(+)ILC1 was found to be decreased in HIV patients whereas numbers of CD103(+)ILC1 and ILC2, respectively, did not differ significantly between HIV(-) controls and HIV patients (Fig 5D, upper panels).

Next, we tested the functional capacity of intestinal ILCs in HIV infection. Following stimulation with PMA/ionomycin IFN-γ production of CD127(+)ILC1 and CD103(+)ILC1 did not differ significantly between HIV-infected individuals and HIV(-) controls. However, we found significantly increased IL-22 production of HIV ILC3 in the colon, resembling data presented by Kim et al. [30]. Of note, no such differences were observed in ILC3 isolated from the duodenum and the ileum, respectively (Fig 5F).

### Intestinal expression of IL-7 in HIV infection

**Fig 6:**
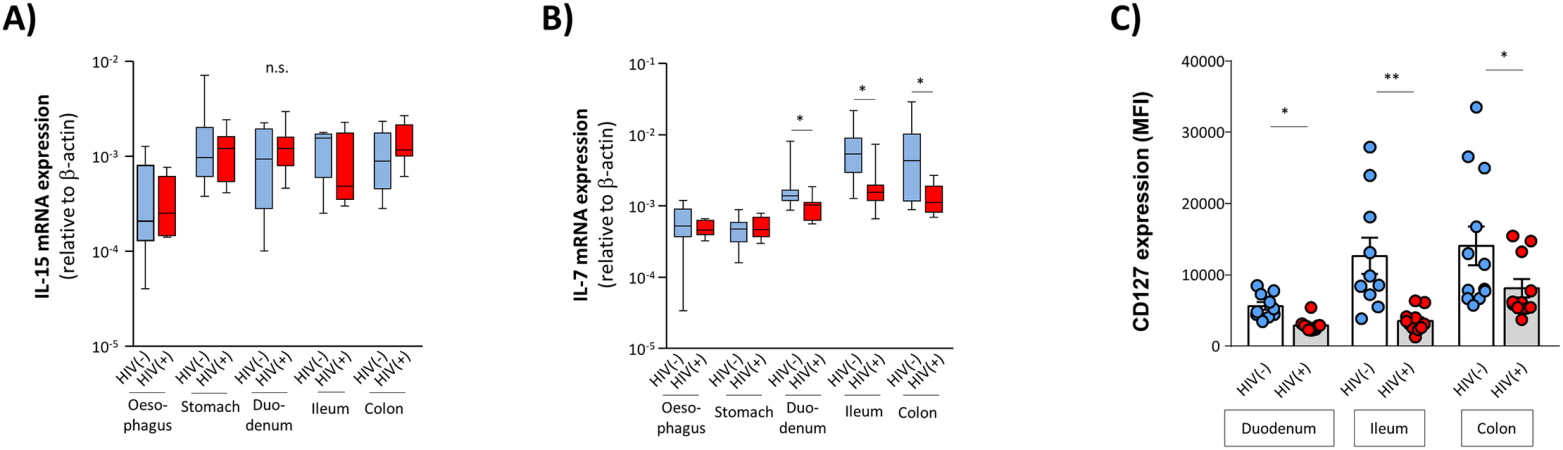
Expression of IL-7mRNA and CD127 in HIV(+) individuals. IL-15 **(A)** and IL-7 **(B)** mRNA expression was studied in the different compartments of the intestinal tract in HIV(+) patients (red) compared to HIV(-) controls (blue). oesophagus: HIV(-) n = 7, HIV(+) n = 6; stomach HIV(-) n = 10, HIV(+) n = 10; duodenum: HIV(-) n = 14, HIV(+) n = 11; ileum: HIV(-) n = 12, HIV(+) n = 15; colon: HIV(-) n = 10, HIV(+) n = 10) **(C)** Surface expression of CD127, expressed as mean fluorescence intensity (MFI), on ILCs isolated from different parts of the GI tract in HIV(+) patients and HIV(-) individuals duodenum: HIV(-) n = 10, HIV(+) n = 10; ileum: HIV(-) n = 10, HIV(+) n = 10; colon: HIV(-) n = 10, HIV(+) n = 11) (* p≤0.05). (FDR-adjusted P values, parametric ANOVA).

**Fig 7:**
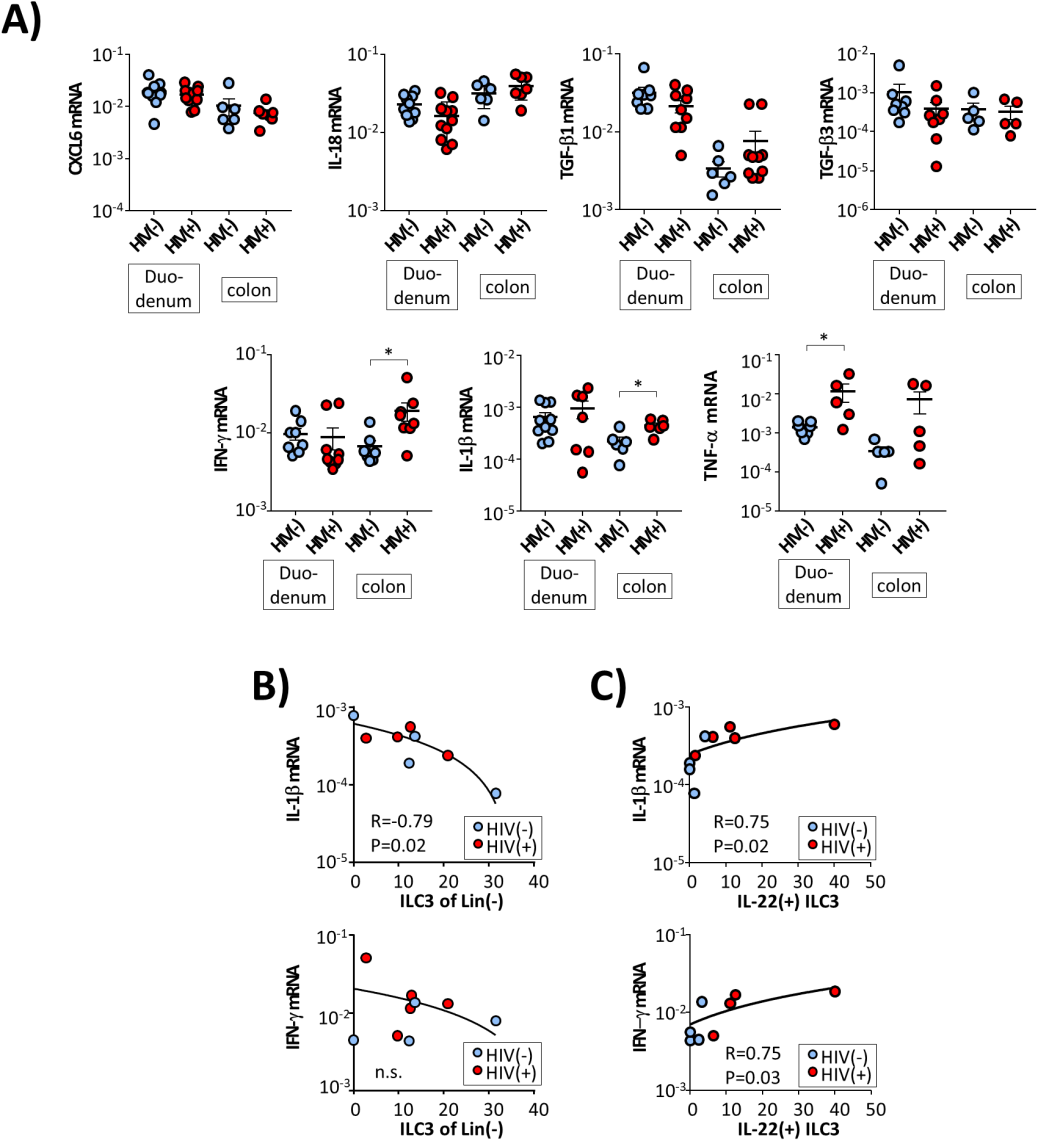
Association between mRNA expression of inflammatory cytokines and frequency as well as functional capacity of the intestinal ILC3 in HIV(+) individuals. **(A)** Gene expression levels of CXCL6, IL-18, TGF-β1/3, IL-1β, IFN-γ, and TNF-α were studied in duodenum and colon samples (* p≤0.05). **(B)** Correlation between IL-1B mRNA expression and frequency of colon ILC3. **(C)** Correlation between IL-1B expression (upper panel) and IFN-γ expression (lower panel) and IL-22 production of colon ILC3.Pearson correlation coefficient was computed for analysing correlations.

Next, we analysed whether HIV-induced alterations of the local IL-7 and/ or IL-15 concentrations might play a role in this context. Regarding IL-15 we did not observe any differences between HIV patients and controls (Fig 6A). In contrast, mucosal IL-7 mRNA levels were significantly decreased in HIV patients specifically in those segments of the GI tract with decreased ILC frequencies (Fig 6B). Moreover, we observed HIV infection to be associated with significantly reduced expression of the IL-7 receptor CD127 in all analysed parts of the GI tract (Fig 6C), suggesting that both decreased mucosal concentrations of IL-7 as well as impaired IL-7 responses of ILCs might contribute to incomplete reconstitution of ILCs under effective anti-retroviral therapy. However, our finding of increased frequency of duodenal ILC3 despite low levels of mucosal IL-7 mRNA and reduced expression of CD127 indicates that other factors also may play a role. Therefore, we studied local mRNA levels of additional cytokines that have been associated with ILC3 homeostasis, such as CXCL6, IL-1β, IL-12, IL-18, and IL-23 [21,31] and/ or intestinal inflammation, including IL-1β, TGF-β, IFN-γ, and TNF-α [32–34]. As is shown in Fig 7, we found HIV infection to be associated with significantly increased levels of the pro-inflammatory cytokines IL-1β and IFN-γ, specifically in the colon. These data suggested that HIV-associated ongoing inflammation in the colon mucosa might also affect the local ILC3 pool. In line with this hypothesis we observed frequency of colon ILC3 to be inversely correlated with mRNA levels of IL-1β (Fig 7B), whereby ILC3 frequency did not correlate with IFN-γ transcripts. Moreover, we observed a positive association between expression of these cytokines and IL-22 production of ILC3 (Fig 7C). With respect to mRNA levels of TNF-α we found significantly increased levels in duodenal samples of HIV patients, whereas gene expression levels in the colon did not differ significantly between HIV(-) and HIV(+) individuals. No associations were found between local TNF-α mRNA expression and frequency or function of intestinal ILC3.

No such alterations were observed with respect to CXCL16, TGF-β1/3, and IL-18. IL-23 mRNA expression could be detected in only a minority of the tested specimen whereas IL-12 expression was found in none of the studied biopsies.

### Loss of colonic ILC3 might be associated with gut epithelial damage in HIV infection

**Fig 8:**
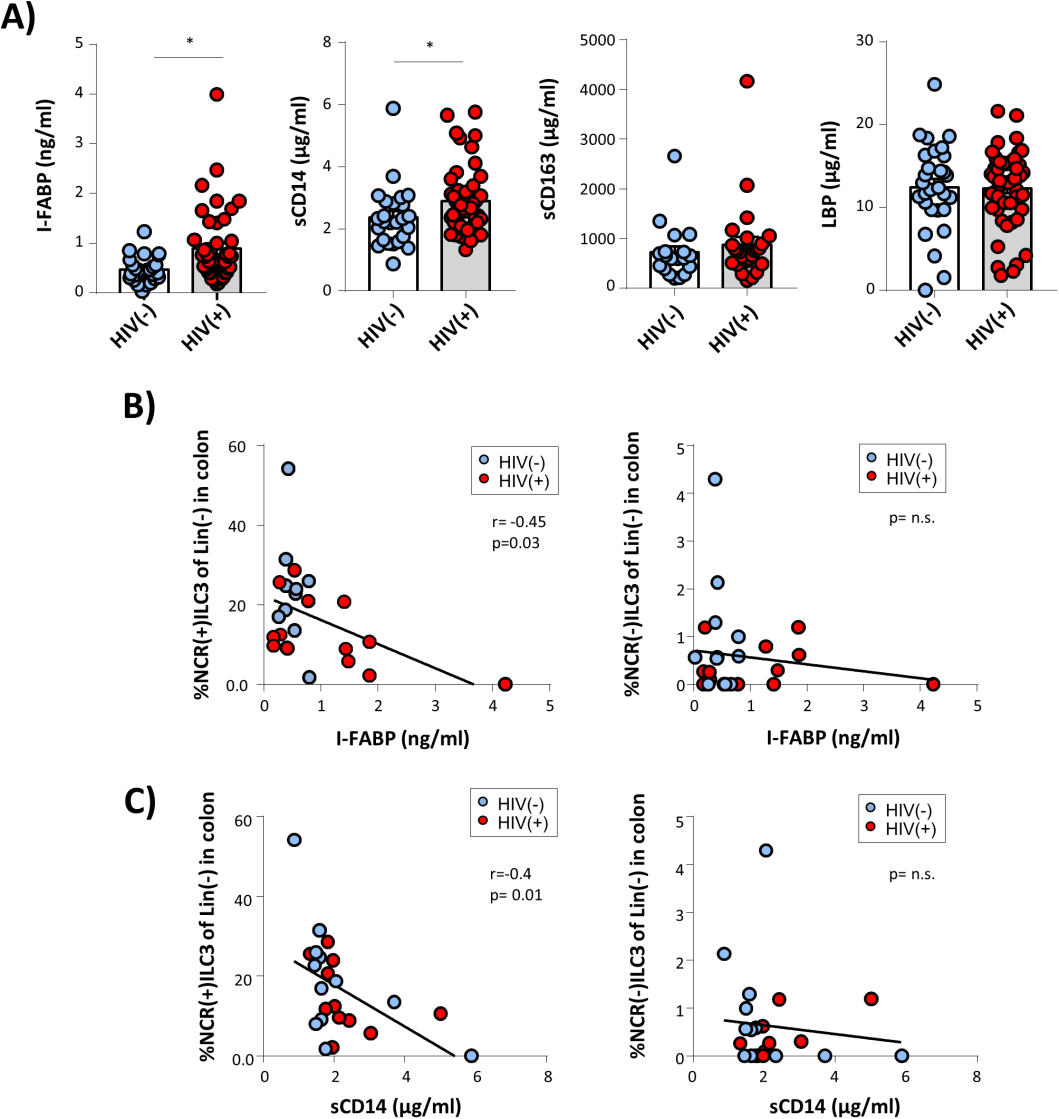
Association between plasma markers of gut epithelial damage/ microbial translocation and colon NCR(+)ILC3 frequency in HIV(+) individuals. **(A)** Plasma serum levels of intestinal fatty acid binding protein (I-FABP; ng/ml), a marker for gut barrier defects, sCD14 (μg/ml), sCD163 (μg/ml), and LPS binding protein (LBP, μg/ml) were quantified by ELISA in HIV patients (red) and HIV(-) controls (blue) (* p≤0.05). Next, association between plasma levels of I-FABP (ng/ml) **(B)** and sCD14 (μg/ml) **(C)**, respectively, and frequency of colon NCR(+)ILC3 (left panel) or colon NCR(-)ILC3 (right panel) were examined. Pearson correlation coefficient was computed for analysing correlations.

HIV-associated damage to the gut epithelial barrier is considered to represent a major mechanism of systemic immune activation even in HIV patients under effective therapy [2,3,35]. As ILCs have been shown to importantly contribute to maintenance of the intestinal epithelial barrier and to promote anatomical containment of commensal bacteria [5], we finally analysed the potential association between HIV-induced dys-regulation of the intestinal ILC pool and gut integrity. In these analyses alterations of the intestinal epithelial barrier were assessed indirectly by measuring the levels of intestinal fatty acid binding protein 1 (I-FABP), a plasma marker that has previously been associated with gut barrier breakdown [36]. In line with this report [36] we found plasma levels of I-FABP to be significantly higher in HIV patients than in controls (Fig 8A). In addition, we observed plasma levels of sCD14, a commonly used indirect marker of microbial translocation, to be significantly increased in HIV(+) patients. More importantly, we observed frequency of colon NCR(+)ILC3 to be inversely correlated with plasma levels of I-FABP (Fig 8B, upper left panel) and sCD14 (Fig 8C, left panel), whereas no such associations were found for colon NCR(-)ILC3 (Fig 8B and 8C, right panels), duodenal ILC3 subsets (S10 Fig) or colon T cell subsets (S9 Fig).

## Discussion

ILCs are widely distributed in the body from hematopoietic organs to secondary lymphoid as well as non-lymphoid tissues. Increasing evidence indicates that localization of ILC subsets in specific tissues relates to their roles in immune and inflammatory responses [13]. Most studies, however, were performed in animal models and focused on one or two ILC subsets. Thus, our knowledge on tissue-specific composition of the human ILC pool is limited.

In the present study, we analysed for the first-time distribution of all currently defined ILC subsets in the complete human GI tract and describe a compartment-specific composition and function of the intestinal ILC pool which is markedly dys-regulated in controlled HIV infection.

At the moment, the exact mechanisms underlying tissue-specific distribution of ILC subsets are only incompletely understood. However, the finding that only few mature non-NK ILCs can be found in bone marrow or fetal liver suggest that the majority of peripheral ILCs originate from ILC progenitors or precursors that emigrated the bone marrow [13]. In turn, tissue-selective migration of lineage-committed ILC precursors and selective differentiation and expansion of common ILC progenitors in the peripheral tissues are considered as key factors in determining ILC distribution in the body.

The local cytokine environment plays an important role in this context. Especially cytokines of the common γ-chain (γc) family have been shown to be essential for the development of all known ILC subsets, with IL-7 being critical for the development of ILC2 and ILC3 subsets and IL-15 playing an important role in the development of cNK cells and CD127(+)ILC1 [37,22,23,38]. Accordingly, our data indicate that compartment-specific concentrations of IL-7 may modulate the intestinal ILC pool as we found mucosal mRNA levels of IL-7 to be positively correlated with frequency of ILC3, whereas no such associations were found concerning group 1 ILCs. With respect to cNK cells we observed an inverse correlation with IL-7 mRNA levels which resembles earlier reports demonstrating IL-7 to be dispensable for cNK cell differentiation [23].

Currently, it remains unclear at what stage IL-7 exerts its effects on ILC homeostasis. Data obtained in mouse models indicate that all ILCs, including cNK cells, are derived from common lymphoid progenitors (CLP) characterized by a Lin(-) c-kit(int)Sca-1(+)CD127(+) phenotype. CLP can differentiate into a Lin(-) Id2(+)CD127(+)CD25(−)α4β7(+)Flt3(−) progenitor population called common helper-like innate lymphoid cell progenitor (CHILP), capable of developing into all ILC subsets except cytotoxic cNK cells, which are believed to branch out from CLP prior to differentiation of CLP to CHILP [37]. Thus, both CLP and CHILP as well as mature ILCs express the IL-7 receptor CD127, suggesting that IL-7 might be required at both the earliest progenitor stages as well as to maintain the respective cell lineages in the tissues. In line with these data Scoville et al. recently identified a human RORgt(+)CD34(+)CD45RA(+)CD117(+)IL-1R1(+) common innate lymphocyte progenitor that capable of generating ILCs when cultured in the presence of IL-7 [39].

Besides IL-7, a variety of cytokines have been shown to regulate ILC differentiation and plasticity. For instance, IL-12 has been shown to drive functional plasticity of human group 2 innate lymphoid cells [40] and to induce a change of ILC3 into c-Kit(-) NKp44(-)ILC1. Moreover differentiation of ILC1 to ILC3 is driven by IL-23 and accelerated by IL-1β, whereas IL-18 can promote differentiation of human ILC3 to ILC1 *in vitro* [21]. Thus, cytokine-mediated effects on the ILC pool are multifaceted and complex, which might also explain why we failed to detect any association between intestinal IL-15 mRNA levels and ILC frequencies.

Moreover, increasing data indicate a complex relationship between the commensal microbiota and development and diversity of the intestinal ILC pool [5,41–43]. In this context, it is important to note that the composition of the mucosal microbiome differs between the compartments of the human GI tract even in healthy persons [44,45].

Thus, it is tempting to speculate that compartment-specific differences in the mucosal microbiome might importantly modulate composition of the local ILC pool. Unfortunately, numbers of biopsies that were available for our study was too low to enable analysing commensal microbiota.

With respect to HIV infection Kløverpris and co-workers recently demonstrated acute disease to be associated with depletion of circulating ILCs which was found to be prevented only by initiation of anti-retroviral therapy during early infection [18]. In line with these data, we observed a trend towards decreased frequencies of peripheral blood ILCs in HIV(+) patients. However, these differences were not statistically significant, which might in part be explained by the lower number of patients analysed in our study. Accordingly, we found HIV infection to be associated with significantly decreased frequencies of ILC3 after including an additional cohort of 37 HIV(-) controls as well as 10 HIV(+) patients (S8 Fig).

More importantly, we observed a significant dys-regulation of the intestinal ILC pool in HIV patients under cART with decreased frequencies of total ILCs especially in the colon. A more detailed analysis demonstrated that this was mainly attributable to reduced numbers of CD127(+)ILC1 and ILC3. Loss of colon ILCs, especially the ILC3 subset, in HIV patients is well in line with data presented recently by Zhang and co-workers [17]. Moreover, rapid and persistent depletion of IL-17-producing ILCs in the GI tract following SIV infection has been shown in previous studies [15,16], suggesting loss of intestinal ILCs to represent a characteristic feature of HIV infection. In extension of these findings we here demonstrate that HIV-associated dys-regulation of the intestinal ILC pool is compartment- and subset-specific. First, we show that neither in the oesophagus nor in the stomach HIV had any significant effect on frequency of the studied ILC subsets. Second, we observed that in contrast to colon ILC3 frequencies were increased in the duodenum of HIV patients. Third, we demonstrate that this increase was specific to the ILC3 subset as frequency of CD127(+)ILC1 was found to be decreased. In addition, we show that frequencies of intestinal CD103(+)ILC1 and ILC2, respectively, did not differ significantly between HIV(-) controls and HIV patients. Moreover, we demonstrate that HIV-associated alterations of the intestinal cell pool is lymphocyte subpopulation-specific as we did not observe any significant changes in cNK cell frequencies, whereas numbers of CD4(+) T cells were reduced in duodenum, ileum as well as colon. Finally, our data indicate that also ILC function is altered in a compartment-specific fashion as we found significantly increased IL-22 production of ILC3 in the colon mucosa of HIV(+) patients compared to HIV(-) controls, whereas no such differences were observed in ILC3 isolated from the duodenum or the ileum.

The mechanisms underlying incomplete restoration of the intestinal ILC pool in patients with controlled HIV infection remain unclear. With respect to CD4(+) T cells it has been suggested that ongoing viral replication as well as chronic immune activation may have detrimental effects on cell substrates and homeostatic microenvironments resulting in a decline of the regenerative potential over time. For instance, HIV infection has been shown to perturb T cell responses to IL-7 via down-modulation of the IL-7 receptor, thereby compromising the regenerative capacity of the T-cell pool [46–49]. In line with these data, we observed HIV infection to be associated with significantly reduced expression of CD127 in all analysed parts of the GI tract. Moreover, we found HIV(+) patients to display significantly decreased levels of mucosal IL-7 mRNA especially in those parts of the GI tract with reduced ILC frequencies, suggesting that impaired IL-7 responses of ILCs might contribute to incomplete reconstitution of ILCs under effective anti-retroviral therapy. In this context, it is important to note that Vanessa and colleagues showed IL-7 to reduce levels of spontaneous apoptosis in CD4(+) and CD8(+) T cells from HIV-1-infected individuals [50]. Given the fact that increased apoptosis has been shown to contribute to loss of colon ILC3 in a mouse model of replicating HIV infection [17] similar mechanisms might also be relevant with respect to human ILCs.

However, our finding of increased frequency of duodenal ILC3 despite reduced expression of CD127 indicates that other factors also may play a role. Whether this includes abnormal JAK/STAT signalling as has been shown for T cells in HIV infection [46,47] remains to be studied.

Our finding of reduced IL-7 levels in HIV(+) is in contrast to data by Napolitano et al. who demonstrated HIV infection to be associated with increased production of IL-7 in lymphocyte-depleted tissues and proposed that increase in IL-7 production might represents a homeostatic response to T-cell depletion [51]. However, these authors studied IL-7 levels in circulating blood and lymph nodes, which might not reflect the situation observed in the intestinal mucosa [52]. Indeed, intestinal epithelial cells have been shown to produce IL-7 [53,54] and we found mRNA expression of villin-1, a marker expressed in intestinal epithelial cells but not in lymphocytes [55–57] to resemble the expression pattern of IL-7 mRNA. Moreover, locally produced IL-7 has been suggested to serve as a potent regulatory factor for intestinal mucosal lymphocytes [53]. Accordingly, IL-7 administration to cART-treated HIV(+) individuals has been shown to improve the gut mucosal barrier [58]. In this study, the authors did not examine ILCs, but linked improved gut barrier function with significant increases in GALT T-cell subsets, which also express CD127.

At the moment the mechanisms involved in dys-regulated intestinal IL-7 expression in HIV(+) patients remains to be clarified. However, increased IL-1β concentrations may play a role in this context as Thang and co-workers demonstrated IL-1β to reduced IL-7 production by stromal and epithelial cells [59].

ILCs have been shown to be required to maintain an intact gut epithelial barrier and to promote the anatomical containment of lymphoid-resistent commensal microbiota [5]. Accordingly, dys-regulation of the ILC pool has been suggested to contribute to HIV-induced impairment of gut-associated lymphoid tissue structure and function [18,60]. In line with this hypothesis, we found frequency of colon NCR(+)ILC3 to be inversely correlated with serum levels of I-FABP, a marker for gut barrier breakdown, suggesting that HIV-induced loss of NCR(+)ILC3 might contribute to microbial translocation, thereby promoting HIV-associated systemic immune activation.

In summary, our data indicate a compartment-specific distribution of ILCs in the human GI-tract. Furthermore, our data suggest a link between markedly dys-regulated intestinal ILCs and loss of intestinal barrier function in HIV infection, thereby allowing for bacterial transmigration and systemic immune activation.

## Materials and methods

### Ethics statement

Written informed consent was obtained from all individuals. The study had been approved by the local ethics committee of the Medical Faculty, University of Bonn (#275/13 and #040/16).

### Patients

A total of 40 HIV-infected individuals (HIV Outpatient Clinic at the University Hospital of Bonn, Germany) as well as 57 HIV(-) controls were enrolled into this study, including patients that underwent colonoscopy for colon cancer screening as well as individuals receiving esophagogastroduodenoscopy (EGD) because of episodes of dysphagia, nausea or abdominal pain.

All HIV(+) patients were under cART and displayed HIV RNA levels below the level of detection. Patient characteristics are given in S1 Table.

### Lymphocyte isolation from tissue specimens

All biopsy samples obtained during routine endoscopy were free of inflammatory or lymphoproliferative bowel diseases on histopathologic examination. Isolation of ILCs from endoscopic biopsies was performed based on recently published protocols [8]. In brief, biopsy samples were cut into small pieces and incubated in Ca^++^/Mg^++^-free Hanks Balanced Salt Solution (GIBCO, Germany) containing 154 μg/ml DTT (Dithiothreitol; Sigma Aldrich, Germany), 5 mM EDTA (AppliChem, Germany), 0.25% NAC (N-acetylcysteine; Sigma, USA) and 1% penicillin/streptomycin (PAN Biotec, Germany) for 45 min at 37°C to dissolve epithelial tight junctions and eliminate mucus. Following centrifugation (50g for 3 min) supernatant was collected and tissue specimens were digested for 45 min at 37°C in RPMI 1640 medium supplemented with 10% FCS and 1% penicillin/streptomycin (complete RPMI medium) containing 1 mg/ml Collagenase II (Worthington, USA) and 25 U/ml Endonuclease (MoBiTec, Germany). Cell suspensions were filtered through a 70μm Nylon cell-strainer (BD, Germany) and washed with PBS.

For isolation of tonsil ILC3, tonsils were cut into small pieces and squeezed through a stainless steel mesh. Single lymphoid cells were isolated using Ficoll-Paque gradient centrifugation (Biochrom AG, Berlin, Germany).

Isolated cells were pre-frozen in freezing medium (complete RPMI medium supplemented with 10% DMSO; Sigma, USA) at -80°C and long-term stored at -150°C.

### PBMC isolation

Human peripheral blood mononuclear cells (PBMCs) were isolated using Ficoll-Paque gradient centrifugation (Biochrom AG, Berlin, Germany). PBMC were pre-frozen in freezing medium at -80°C and long-term stored at -150°C.

### FACS analyses and cell sorting

Phenotypic analyses of cells were performed using a LSR-Fortessa Cytometer (BD, Germany). The antibodies used in these studies are listed in S2 Table. Isotype controls were used in all experiments.

For intracellular analyses of transcription factors the Foxp3 Transcription Factor Staining Kit (eBioscience, Germany) was used for permeabilization, fixation, and washing. For sorting by flow cytometry an ARIA III (BD Biosciences) was used. Data were analyzed with FlowJo software V10.1 (TreeStar).

### Cytokine stimulation

To evaluate effects of recombinant IL-7 (Immunotools, Friesoythe; Germany) on ILC CD127 and RORγt expression, sorted human ILC3 cells from tonsils were co-cultured with increasing concentrations of IL-7 (0, 10, 50 ng/ml) for 72h in complete RPMI medium.

### Intracellular cytokine staining

Cells were stimulated for 4h with phorbol-12-myristat-13-acetat (PMA; Cell Signaling Technology Europe, Netherlands) (35 ng/ml) and ionomycin (Cell Signaling Technology Europe) (1000 ng/ml) in complete RPMI 1640 medium in the presence of brefeldin A (BFA; Enzo, Germany) (5 μg/ml) for the final 2h. Then, cells were washed, stained, and permeabilized using the Cytofix/Cytoperm Kit (BD, Germany). IFN-γ, IL-22 and IL-13 were detected with specific antibodies by intracellular staining. Data were acquired with an LSR-Fortessa Cytometer (BD Biosciences) and analyzed with FlowJo software V10.1 (TreeStar).

A complete list of the antibodies used in this study is given in S2 Table.

### PCR analysis

Tissue specimens were frozen using -80°C cold isopentan and stored at -80°C until analysis. After thawing messenger RNA (mRNA) was extracted using the GeneJet RNA purification kit (Thermo Scientific, Germany) and cDNA was transcribed using the QuantiTect reverse transcription kit (Qiagen, Germany) in accordance to the manufacturer´s protocol. qPCR was performed on a LightCycler (Roche, Germany) with the Maxima SYBR Green master mix (Thermo Scientific) using the primer sets depicted in S3 Table.

Relative expression of the respective target gene is given in relation to beta-actin expression.

### ELISA

Plasma levels of intestinal fatty acid binding protein (iFABP) (Hycultec, Germany), sCD14 (R&D, Germany), CD163 (ebioscience, Germany), and LBP (Hycultec, Germany) were measured by ELISA following the manufacturers´ protocols.

### Statistical analysis

Compartment-specific frequencies of intestinal cell sub-populations in HIV(-) controls and between HIV(-) and HIV(+) individuals were compared using parametric ANOVA. Test multiplicity was controlled by a false discovery rate (FDR) procedure accounting for dependency among statistical tests [61]. FDR-adjusted P values <0.05 were considered statistically significant. For correlation analyses Pearson correlation coefficients were computed. Statistical analyses were performed using GraphPad Prism Version 5.0a (GraphPad Software Inc, San Diego, CA, USA) and the SPSS 20.0 software package (IBM, Germany). Figures were created using GraphPad Prism 5.0 (GraphPad Software, Inc.; USA).

## Supporting information

S1 Fig Gating strategy used for the identification of ILC subsets in the different parts of the GI tract. (TIF)

S2 Fig Frequency of ILCs in relation to CD45(+) cells. Distribution of CD103(+)ILC1 (upper left panel), CD127(+)ILC1 (upper middle panel), ILC2 (upper right panel), total ILC3 (lower left panel) as well as NCR(+) and NCR(-) ILC3 subsets in the different segments of human intestinal tract (black columns) and the circulating blood (white columns). Frequency of ILCs is given in relation to CD45(+) cells.
of CD103(+)ILC1: PBMC, n.d.; oesophagus, n = 12; stomach n = 15; duodenum, n = 21; ileum, n = 15; colon: n = 13
CD127(+)ILC1: PBMC, n = 19; oesophagus, n = 15; stomach n = 16; duodenum, n = 31; ileum, n = 18; colon: n = 18
ILC2: PBMC, n = 19; oesophagus, n = 15; stomach n = 16; duodenum, n = 31; ileum, n = 18; colon: n = 18
ILC3: PBMC, n = 19; oesophagus, n = 15; stomach n = 17; duodenum, n = 30; ileum, n = 19; colon: n = 19.
(TIF)

S3 Fig ILC2 represent a minor ILC population in the human gut. **(A)** Intra-epithelilal and lamina propria lymphocytes were prepared following previously described protocol (Bernink JH, Krabbendam L, Germar K, et al. Interleukin-12 and -23 Control Plasticity of CD127(+) Group 1 and Group 3 Innate Lymphoid Cells in the Intestinal Lamina Propria. Immunity 2015; modification: instead Liberase TM we have used 1mg/ml Collagenase Type II by Worthington/USA). Then ILC frequency was studied by FACS analysis using the gating strategy depicted in Fig 1, which confirmed that ILC2 represent a minor ILC population in the human gut. **(B)** In order to exclude that the digestion protocol used for isolation of intestinal ILCs might interfere with expression of the ILC2 marker CRTH2, we compared CRTH2 expression on circulating Lin(-)CD45(+)CD161(+)CD127(+) that were cultured in the presence (“with treatment“) or abscence (“w/o treatment“) Collagenase II (1 mg/ml) and Endonuclease (25 U/ml) for 45min at 37°C.
(TIF)

S4 Fig Functional capacity of NCR(+) and NCR(-) ILC subsets. **(A)** Gating strategy used for identification of NCR(+) and NCR(-) ILC subsets. ILCs were stimulated by PMA/ionomycin. Then, cytokine production of NCR(+) and NCR(-) subsets was studied. A representative sample is given in **(B)**. **(C)** Functional capacity of NCR(+) and NCR(-) ILC subsets isolated from different parts of the human GI tract.
(TIF)

S5 Fig Local IL-7 levels and composition of the intestinal ILC pool in HIV(-) individuals. **(A)** Compartment-specific IL-7mRNA levels (green) in comparison to frequency of total ILCs (orange; left panel; oesophagus (n = 6), stomach (n = 7), duodenum (n = 10), ileum (n = 9), and colon (n = 8)) and cNK cells (brown; right panels; oesophagus (n = 6), stomach (n = 7), duodenum (n = 9), ileum (n = 9), and colon (n = 7)) in the different segments of the GI tract. The right panel depicts correlation between local IL-7 mRNA levels and ILC frequency. **(B)** Local IL-7 mRNA concentrations (green) in relation to frequency of CD127(+)ILC1 (blue; upper left panel), CD103(+)ILC1 (coppery; upper right panel), ILC2 (green; lower left panel), and ILC3 **(C)**(violet; lower right panel). oesophagus (n = 6), stomach (n = 7), duodenum (n = 10), ileum (n = 9), and colon (n = 8). The right panel depicts correlation between local IL-7 mRNA levels and ILC3 frequency. Frequency of ILCs is given in relation to CD45(+) cells.
(TIF)

S6 Fig Local IL-7 levels and composition of the intestinal ILC pool in HIV(-) individuals. **(A)** Mucosal cytokine/ chemokine mRNA expression levels were analysed in the oesophagus (n = 7), the stomach (n = 9), the duodenum (n = 10), the ileum (n = 9), and the colon (n = 7), respectively. **(B)** Compartment-specific cytokine mRNA levels in comparison to frequency of total ILCs and ILC subsets in the different segments of the GI tract (oesophagus (n = 6), stomach (n = 7), duodenum (n = 10), ileum (n = 9), and colon (n = 7)). Cytokine mRNA expression levels are expressed in relation to expression levels of b-actin. Frequency of ILCs is given in relation to Lin(-) cells.
(TIF)

S7 Fig Alterations of the intestinal ILC pool in HIV(+) individuals. **(A)** Frequency of total ILCs in the different segments of the GI tract in HIV(-) controls compared to HIV patients (* p≤0.05). **(B)** Frequency of CD103(+)ILC1 (upper left panel), ILC2 (upper right panel), CD127(+)ILC1 (lower left panel), and ILC3 (lower right panel; in the different segments of the GI tract in HIV(-) controls compared to HIV patients. Frequencies of total ILCs and ILC subsets are expressed in relation to CD45(+) cells.
(TIF)

S8 Fig Frequency of circulating ILCs in HIV(+) under effective cART. Frequencies of circulating ILCs were studied in 19 HIV(+) patients and 20 healthy controls in which both blood as well as tissue samples were availble. These analyses revealed a tendency towards decreased numbers of peripheral ILC2 and ILC3, respectively, in HIV(+) patients compared to HIV(-) controls. However, these differences did not reach statistical significance. This was surprising as Kløverpris and colleagues recently demonstrated significantly decreased frequencies of circulating ILCs even in HIV(+) patients under effective therapy. To clarify whether this discrepancy might be explained by a lower number of patients analysed in our study, we included a additional cohort of 37 healthy controls (pale blue) as well as 10 HIV(+) patients under cART (pale red). Indeed, we found that after increasing sample size frequency of ILC3 was significantly lower than in controls. However, no such differences could be confirmed for the other subsets or total ILCs. Sample size calculation (http://www.openepi.com/SampleSize/SSMean.htm) revealed that more than 110 samples in each group would be needed to detect a difference in mean frequency of total ILCs and ILC2, respectively, with a power of 80% (CI 95%).
(TIF)

S9 Fig Intestinal T cells and gut epithelial integrity. **(A)** Frequency of IL-22(+) T cells following stimulation with PMA/ionomycin. **(B)** CD127 expression (MFI) on IL-22 (+) T cells between HIV(-) and HIV(+) individuals. **(C)** Correlation between IFABP (left panels) or sCD14 (right panels) with % of IL-22(+) T cells (upper panels), % of CD3+ T cells (intermediate panels) and % of CD4+ T cells (lower panels), respectively, in the colon.
(TIF)

S10 Fig Association between plasma markers of gut epithelial damage/ microbial translocation and duodenum NCR(+)ILC3 frequency. Plasma levels of I-FABP (ng/ml) and sCD14 (μg/ml), respectively, were analysed and compared to frequency of duodenum NCR(+)ILC3 (left panel) or duodenum NCR(-)ILC3 (right panel). Pearson correlation coefficient was computed for analysing correlations.
(TIF)

S1 Table Patient characteristics. (DOCX)

S2 Table List of mAbs used in this study in 4 different panels for multicolor flow cytometry. (DOCX)

S3 Table List of primer sequences used for quantitative PCR. (DOCX)
