# Supplementary figures and images for: Compartment-specific distribution of human intestinal innate lymphoid cells is altered in HIV patients under effective therapy
Source: PLoS Pathog. 2017 May 15;13(5):e1006373. doi: 10.1371/journal.ppat.1006373 (PMC5444854; doi:10.1371/journal.ppat.1006373)

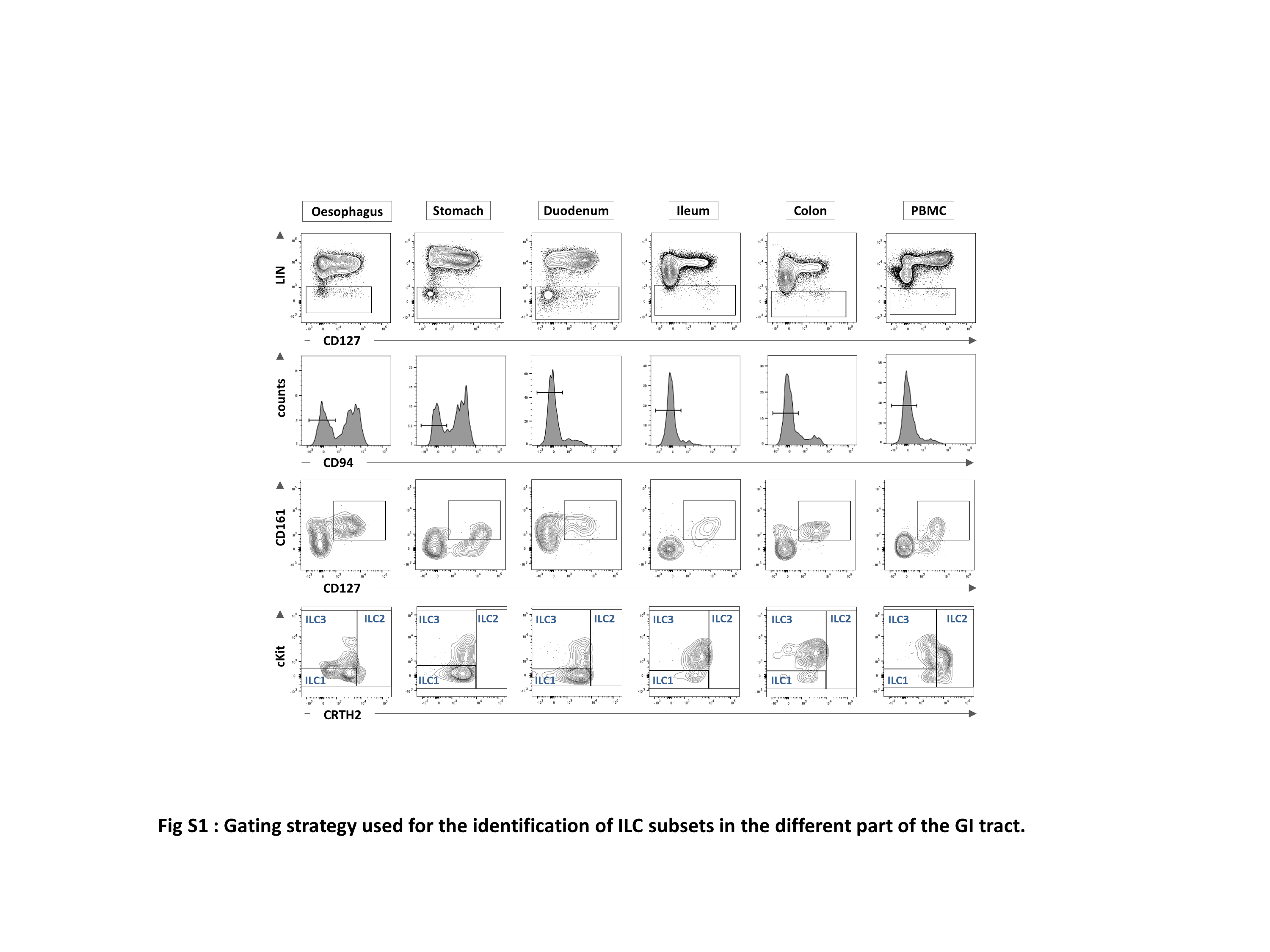

Supplement: S1 Fig — (TIF) [file ppat.1006373.s001.tif]

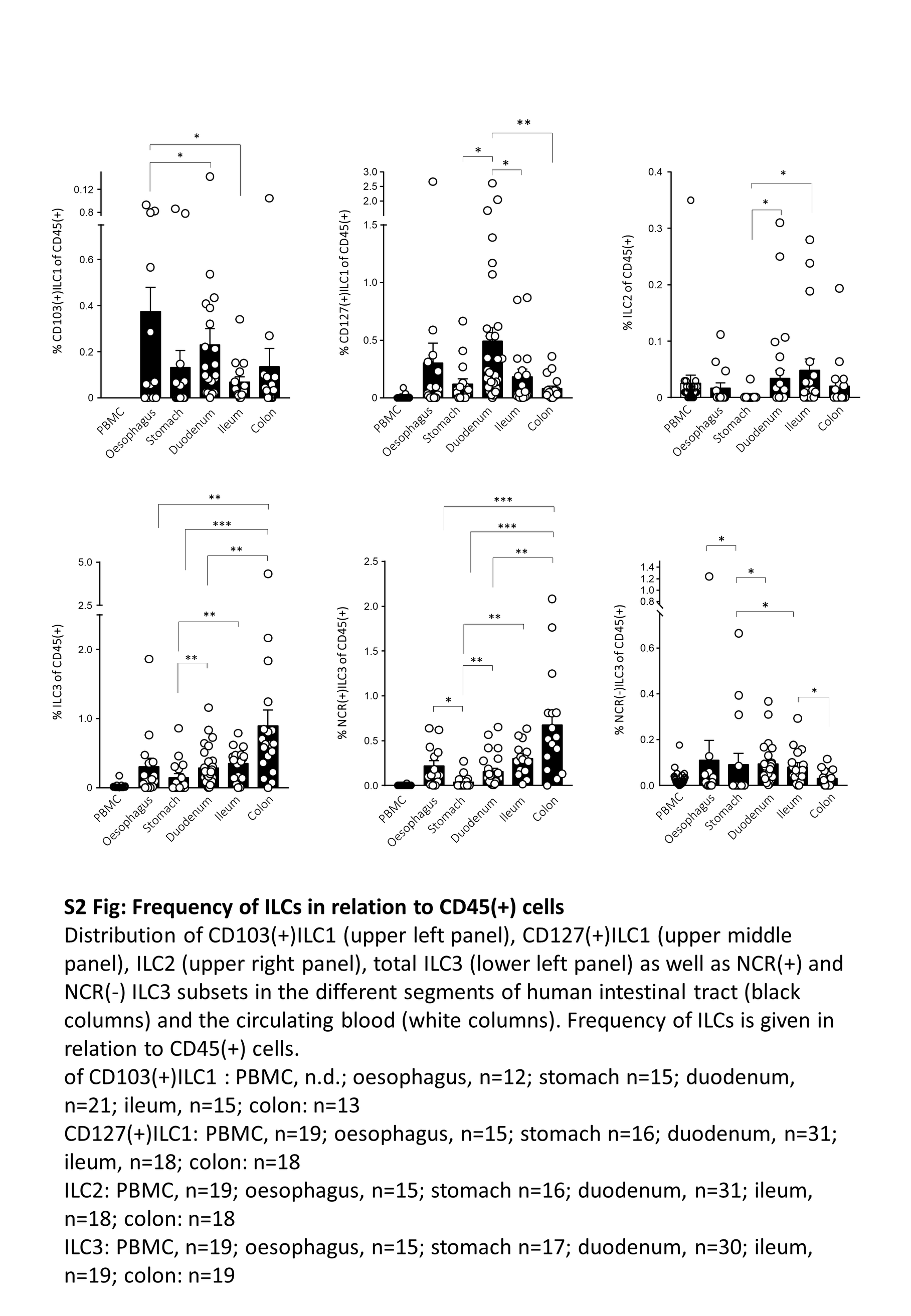

Supplement: S2 Fig — Distribution of CD103(+)ILC1 (upper left panel), CD127(+)ILC1 (upper middle panel), ILC2 (upper right panel), total ILC3 (lower left panel) as well as NCR(+) and NCR(-) ILC3 subsets in the different segments of human intestinal tract (black columns) and the circulating blood (white columns). Frequency of ILCs is given in relation to CD45(+) cells. of CD103(+)ILC1: PBMC, n.d.; oesophagus, n = 12; stomach n = 15; duodenum, n = 21; ileum, n = 15; colon: n = 13 CD127(+)ILC1: PBMC, n = 19; oesophagus, n = 15; stomach n = 16; duodenum, n = 31; ileum, n = 18; colon: n = 18 ILC2: PBMC, n = 19; oesophagus, n = 15; stomach n = 16; duodenum, n = 31; ileum, n = 18; colon: n = 18 ILC3: PBMC, n = 19; oesophagus, n = 15; stomach n = 17; duodenum, n = 30; ileum, n = 19; colon: n = 19. (TIF) [file ppat.1006373.s002.tif]

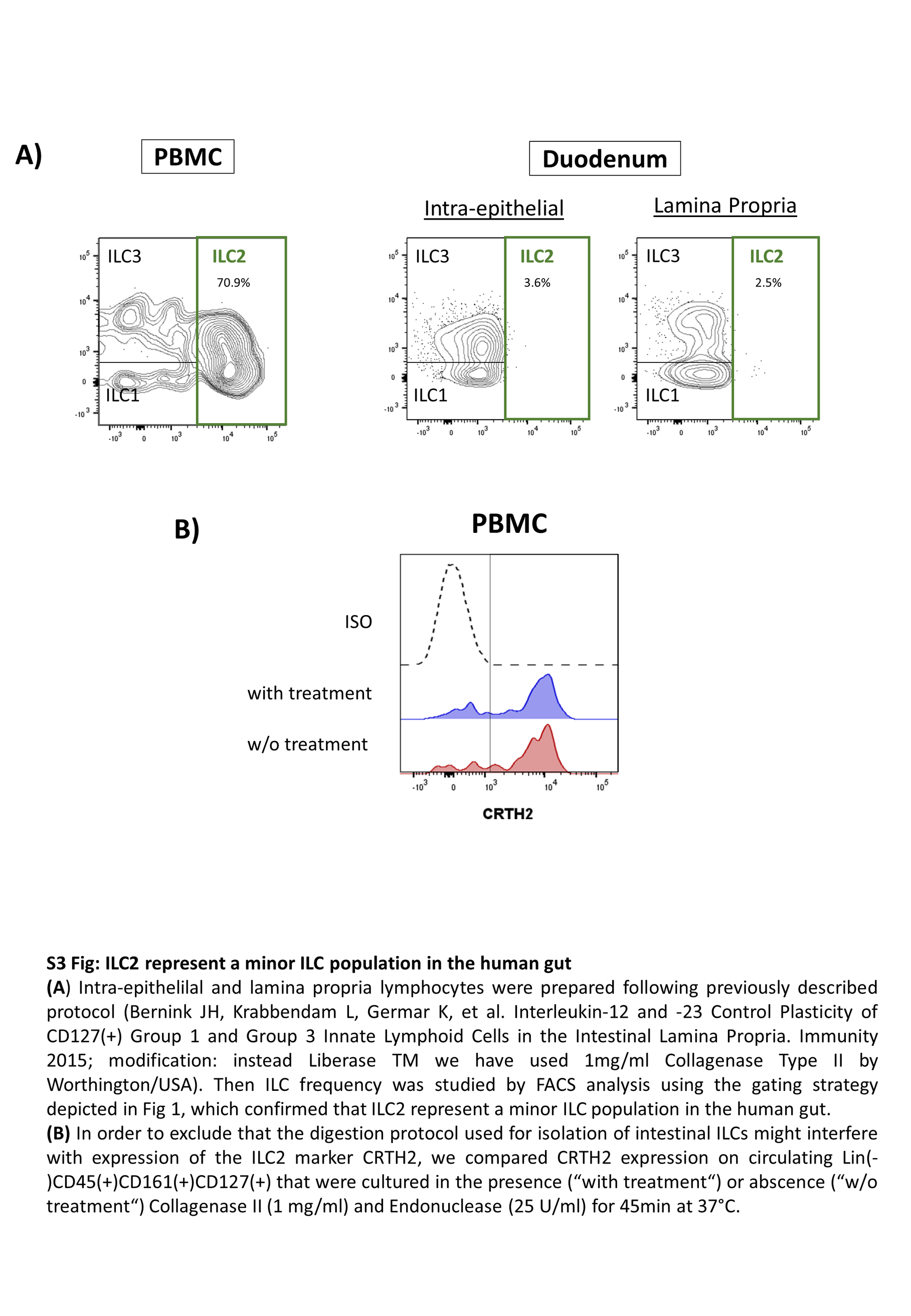

Supplement: S3 Fig — (A) Intra-epithelilal and lamina propria lymphocytes were prepared following previously described protocol (Bernink JH, Krabbendam L, Germar K, et al. Interleukin-12 and -23 Control Plasticity of CD127(+) Group 1 and Group 3 Innate Lymphoid Cells in the Intestinal Lamina Propria. Immunity 2015; modification: instead Liberase TM we have used 1mg/ml Collagenase Type II by Worthington/USA). Then ILC frequency was studied by FACS analysis using the gating strategy depicted in Fig 1, which confirmed that ILC2 represent a minor ILC population in the human gut. (B) In order to exclude that the digestion protocol used for isolation of intestinal ILCs might interfere with expression of the ILC2 marker CRTH2, we compared CRTH2 expression on circulating Lin(-)CD45(+)CD161(+)CD127(+) that were cultured in the presence (“with treatment“) or abscence (“w/o treatment“) Collagenase II (1 mg/ml) and Endonuclease (25 U/ml) for 45min at 37°C. (TIF) [file ppat.1006373.s003.tif]

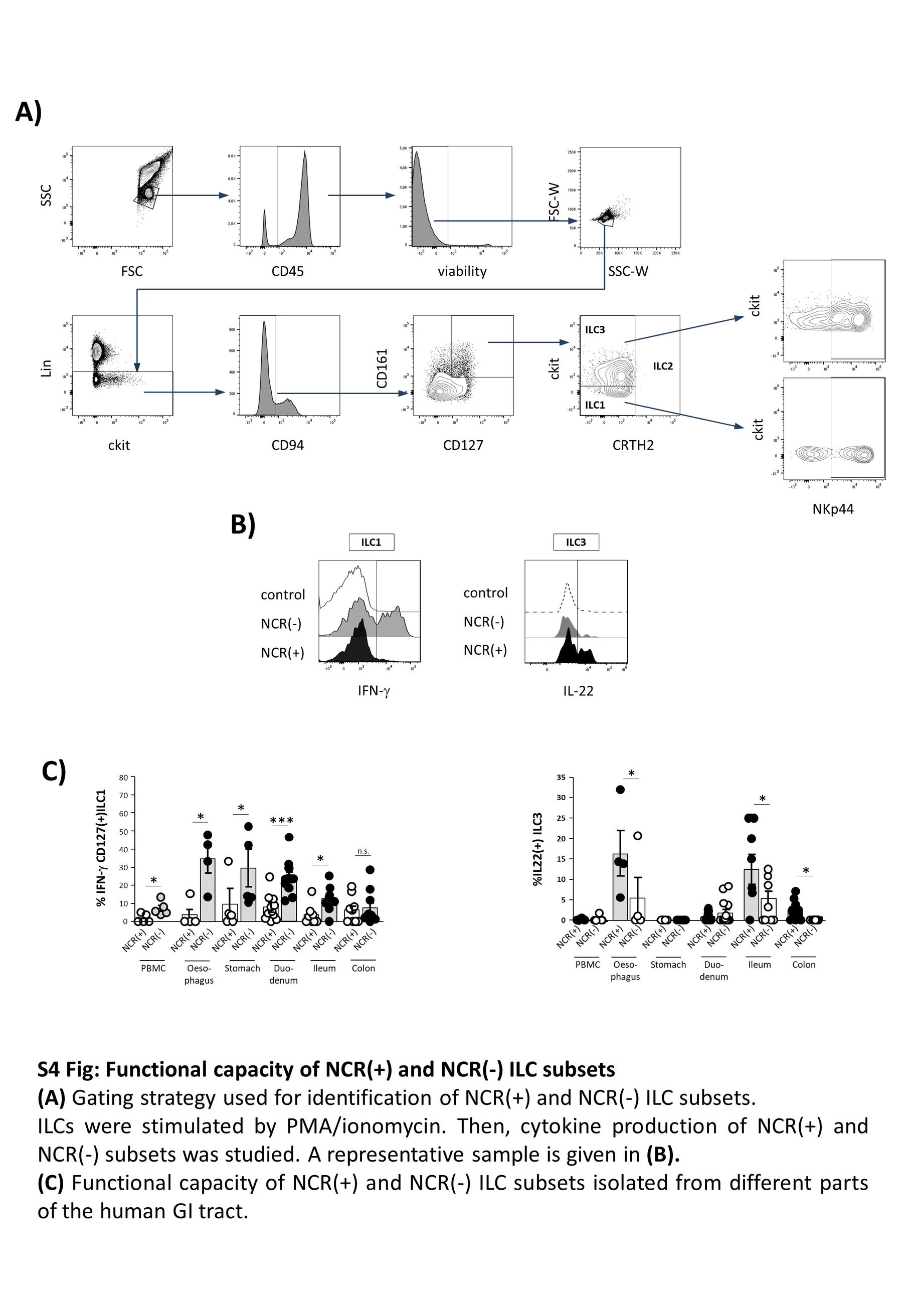

Supplement: S4 Fig — (A) Gating strategy used for identification of NCR(+) and NCR(-) ILC subsets. ILCs were stimulated by PMA/ionomycin. Then, cytokine production of NCR(+) and NCR(-) subsets was studied. A representative sample is given in (B). (C) Functional capacity of NCR(+) and NCR(-) ILC subsets isolated from different parts of the human GI tract. (TIF) [file ppat.1006373.s004.tif]

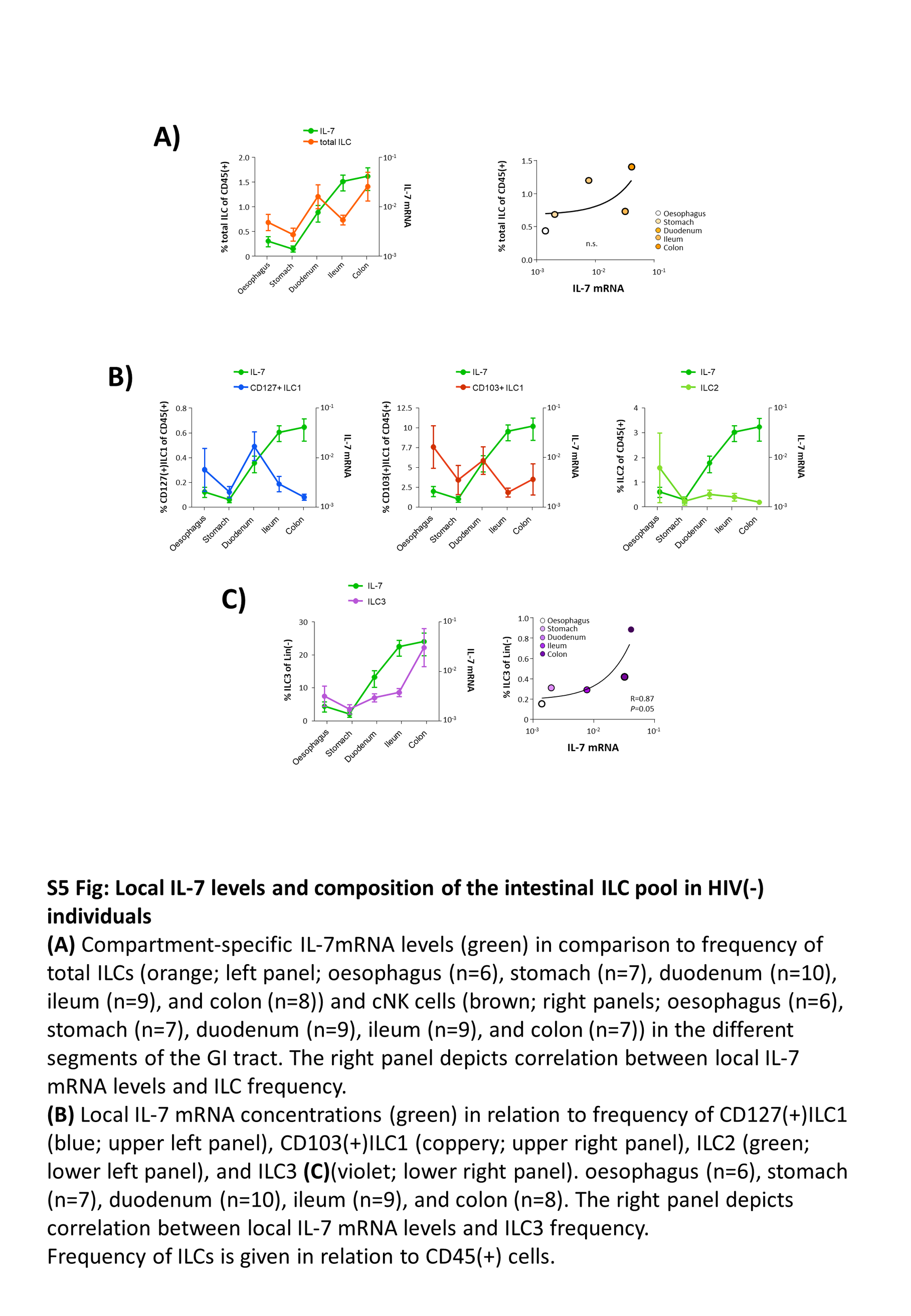

Supplement: S5 Fig — (A) Compartment-specific IL-7mRNA levels (green) in comparison to frequency of total ILCs (orange; left panel; oesophagus (n = 6), stomach (n = 7), duodenum (n = 10), ileum (n = 9), and colon (n = 8)) and cNK cells (brown; right panels; oesophagus (n = 6), stomach (n = 7), duodenum (n = 9), ileum (n = 9), and colon (n = 7)) in the different segments of the GI tract. The right panel depicts correlation between local IL-7 mRNA levels and ILC frequency. (B) Local IL-7 mRNA concentrations (green) in relation to frequency of CD127(+)ILC1 (blue; upper left panel), CD103(+)ILC1 (coppery; upper right panel), ILC2 (green; lower left panel), and ILC3 (C)(violet; lower right panel). oesophagus (n = 6), stomach (n = 7), duodenum (n = 10), ileum (n = 9), and colon (n = 8). The right panel depicts correlation between local IL-7 mRNA levels and ILC3 frequency. Frequency of ILCs is given in relation to CD45(+) cells. (TIF) [file ppat.1006373.s005.tif]

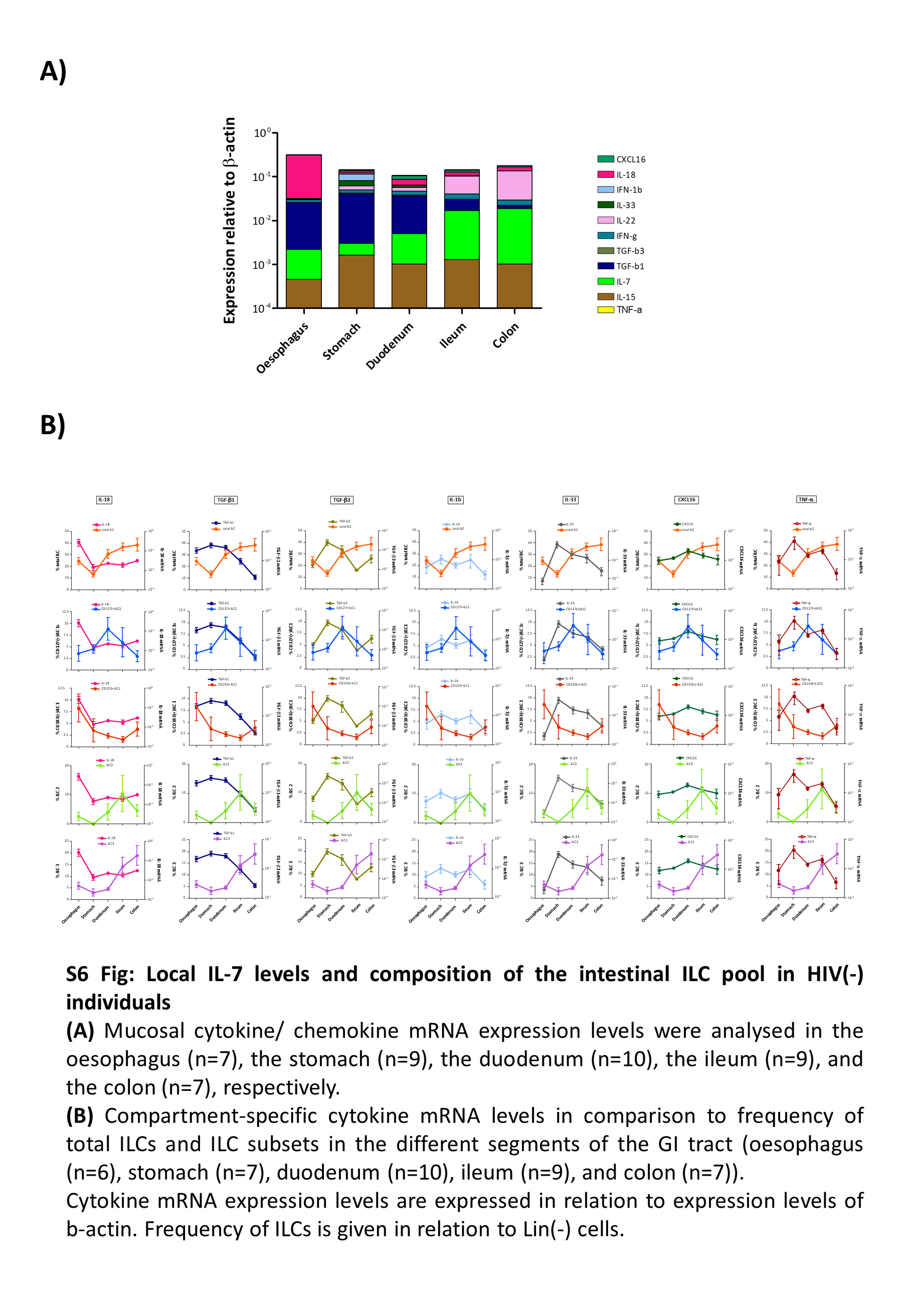

Supplement: S6 Fig — (A) Mucosal cytokine/ chemokine mRNA expression levels were analysed in the oesophagus (n = 7), the stomach (n = 9), the duodenum (n = 10), the ileum (n = 9), and the colon (n = 7), respectively. (B) Compartment-specific cytokine mRNA levels in comparison to frequency of total ILCs and ILC subsets in the different segments of the GI tract (oesophagus (n = 6), stomach (n = 7), duodenum (n = 10), ileum (n = 9), and colon (n = 7)). Cytokine mRNA expression levels are expressed in relation to expression levels of b-actin. Frequency of ILCs is given in relation to Lin(-) cells. (TIF) [file ppat.1006373.s006.tif]

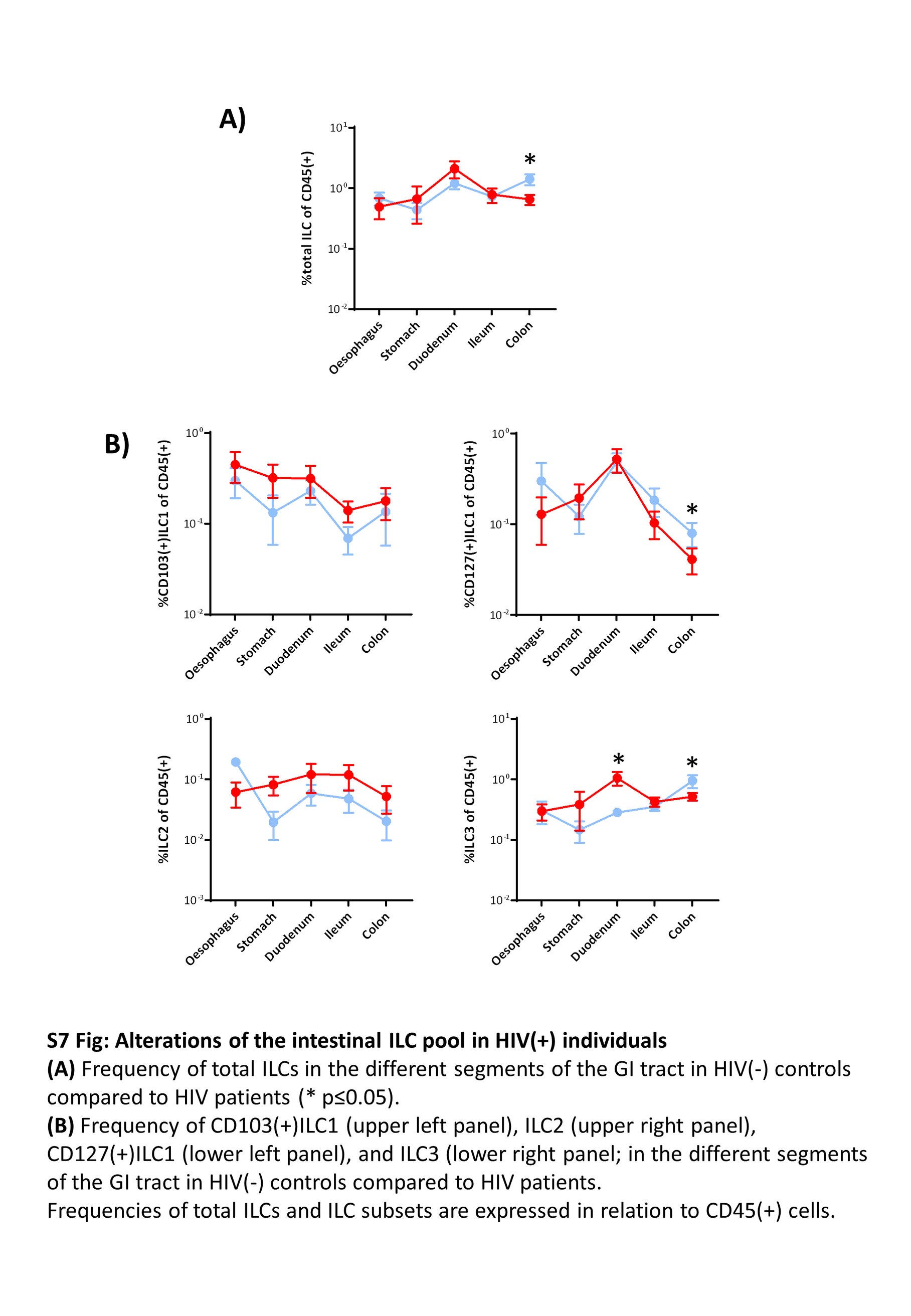

Supplement: S7 Fig — (A) Frequency of total ILCs in the different segments of the GI tract in HIV(-) controls compared to HIV patients (* p≤0.05). (B) Frequency of CD103(+)ILC1 (upper left panel), ILC2 (upper right panel), CD127(+)ILC1 (lower left panel), and ILC3 (lower right panel; in the different segments of the GI tract in HIV(-) controls compared to HIV patients. Frequencies of total ILCs and ILC subsets are expressed in relation to CD45(+) cells. (TIF) [file ppat.1006373.s007.tif]

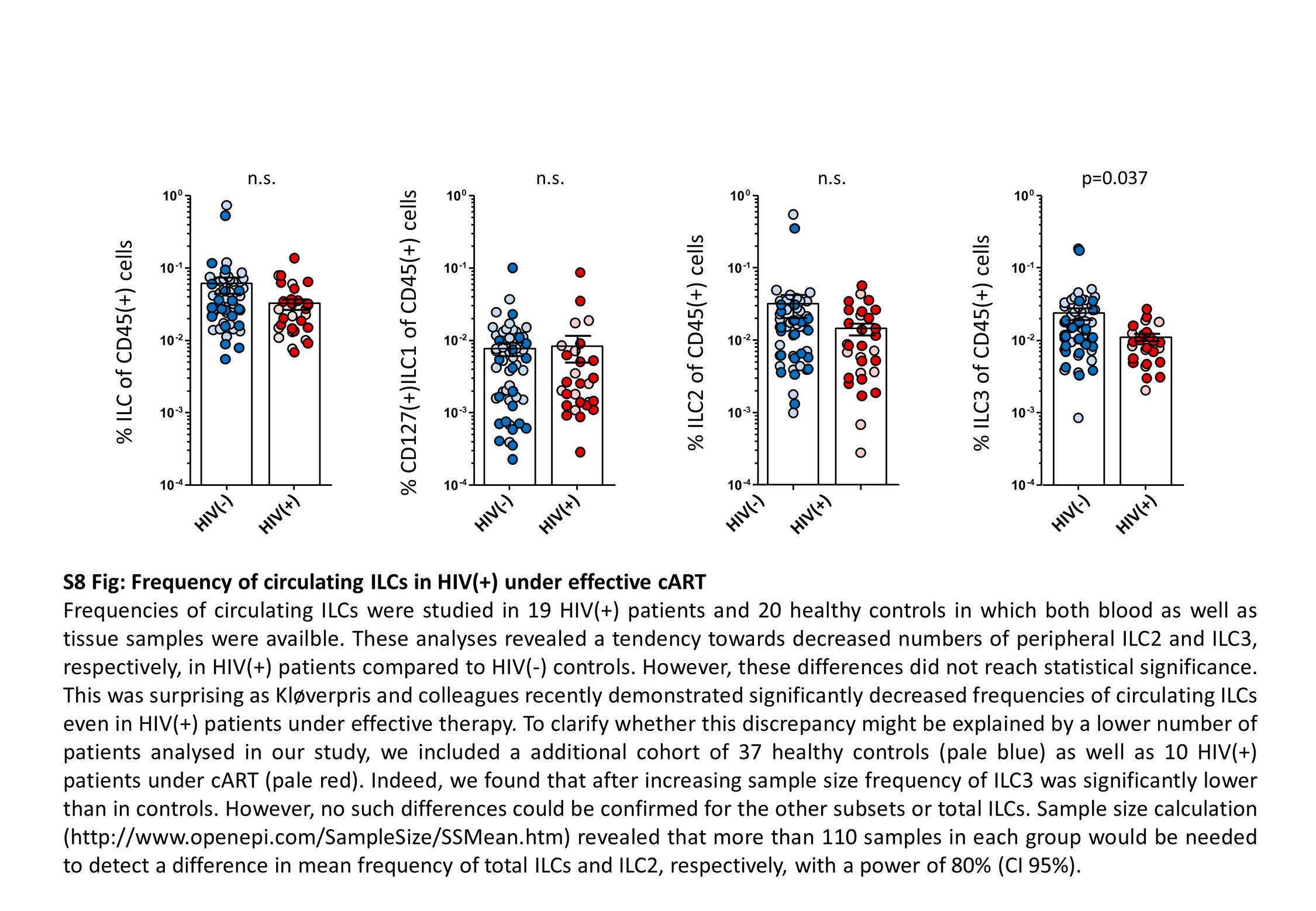

Supplement: S8 Fig — Frequencies of circulating ILCs were studied in 19 HIV(+) patients and 20 healthy controls in which both blood as well as tissue samples were availble. These analyses revealed a tendency towards decreased numbers of peripheral ILC2 and ILC3, respectively, in HIV(+) patients compared to HIV(-) controls. However, these differences did not reach statistical significance. This was surprising as Kløverpris and colleagues recently demonstrated significantly decreased frequencies of circulating ILCs even in HIV(+) patients under effective therapy. To clarify whether this discrepancy might be explained by a lower number of patients analysed in our study, we included a additional cohort of 37 healthy controls (pale blue) as well as 10 HIV(+) patients under cART (pale red). Indeed, we found that after increasing sample size frequency of ILC3 was significantly lower than in controls. However, no such differences could be confirmed for the other subsets or total ILCs. Sample size calculation (http://www.openepi.com/SampleSize/SSMean.htm) revealed that more than 110 samples in each group would be needed to detect a difference in mean frequency of total ILCs and ILC2, respectively, with a power of 80% (CI 95%). (TIF) [file ppat.1006373.s008.tif]

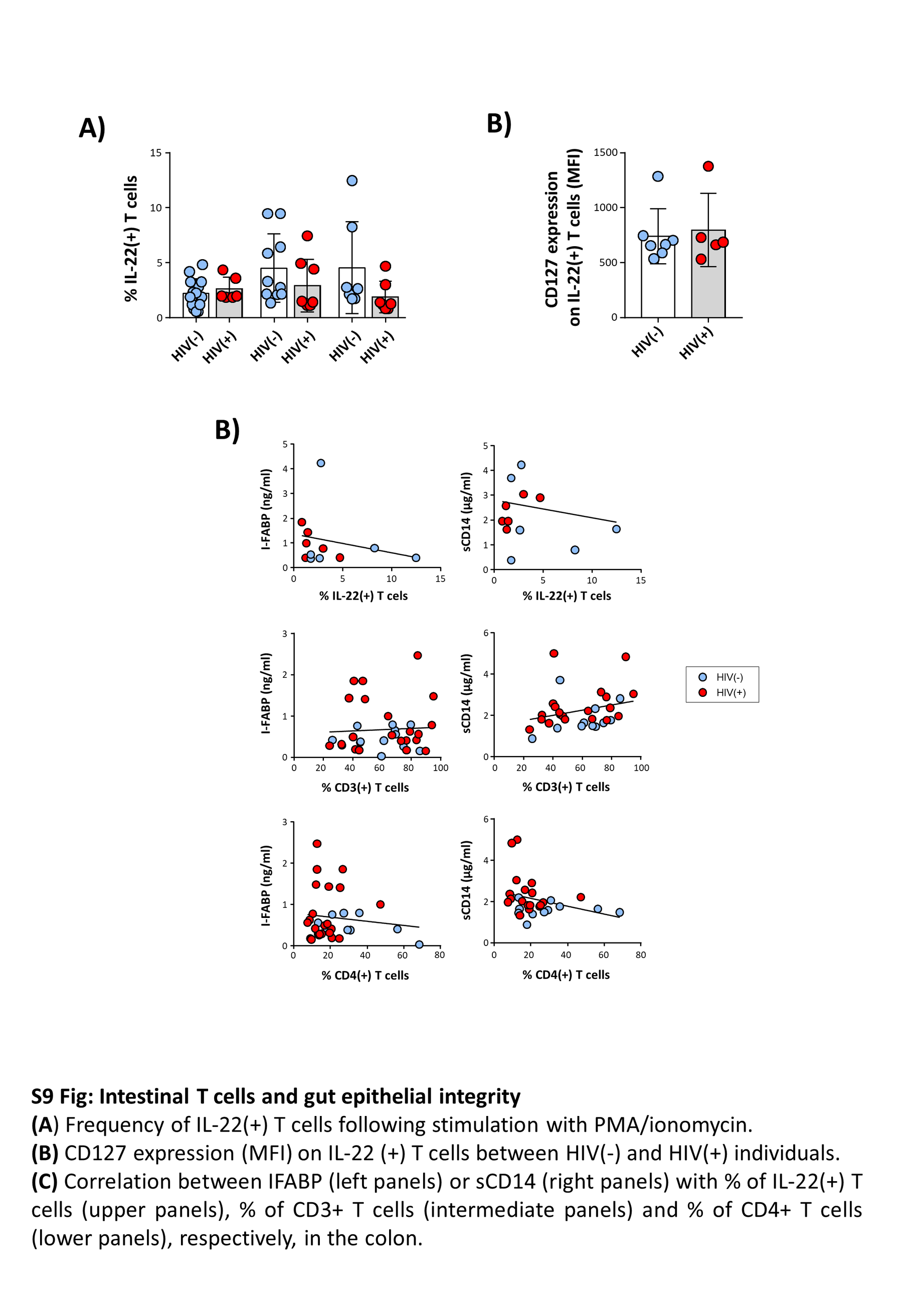

Supplement: S9 Fig — (A) Frequency of IL-22(+) T cells following stimulation with PMA/ionomycin. (B) CD127 expression (MFI) on IL-22 (+) T cells between HIV(-) and HIV(+) individuals. (C) Correlation between IFABP (left panels) or sCD14 (right panels) with % of IL-22(+) T cells (upper panels), % of CD3+ T cells (intermediate panels) and % of CD4+ T cells (lower panels), respectively, in the colon. (TIF) [file ppat.1006373.s009.tif]

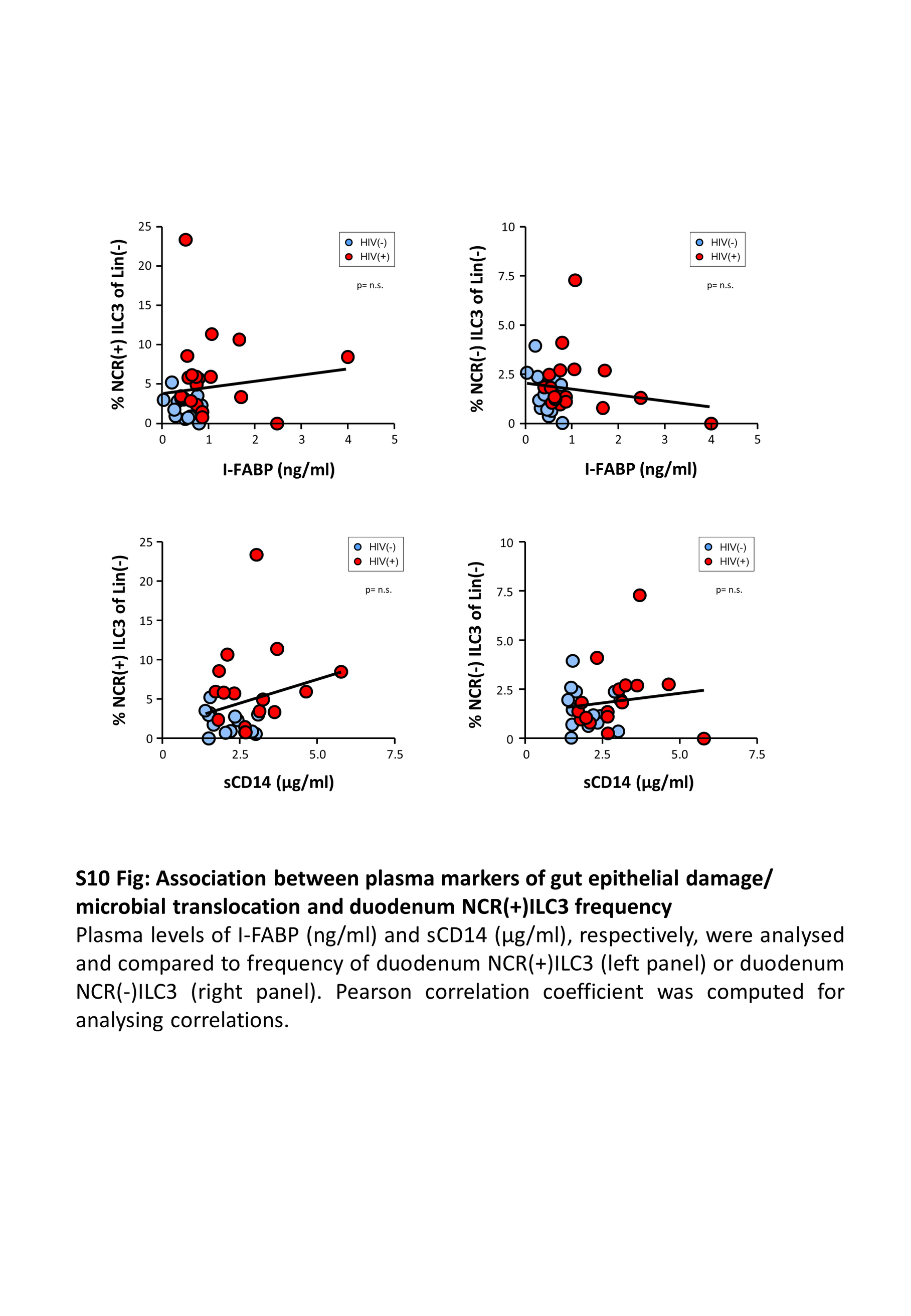

Supplement: S10 Fig — Plasma levels of I-FABP (ng/ml) and sCD14 (μg/ml), respectively, were analysed and compared to frequency of duodenum NCR(+)ILC3 (left panel) or duodenum NCR(-)ILC3 (right panel). Pearson correlation coefficient was computed for analysing correlations. (TIF) [file ppat.1006373.s010.tif]
