# Supplementary material for: Compartment-specific distribution of human intestinal innate lymphoid cells is altered in HIV patients under effective therapy
Source: PLoS Pathog. 2017 May 15;13(5):e1006373. doi: 10.1371/journal.ppat.1006373 (PMC5444854; doi:10.1371/journal.ppat.1006373)
Supplement: S1 Table — (DOCX) [file ppat.1006373.s011.docx]

|  | **HIV(-) controls** | **HIV(+) patients** | **P** |
| --- | --- | --- | --- |
| **Patient number ^1^** | 57 | 40 |  |
| **Male sex ^1^** | 30 (52.6%) | 34 (85%) | <0.001 |
| **Age [years ]^2^** | 52 (19;89) | 51 (28;76) | 0.7 |
| **Body-Mass-Index [kg/m^2^]^2^** | 28.1 (18.2; 34.4) | 25.8 (19.9; 37.1) | 0.32 |
| **Antiretroviral therapy ^1^** | n.a. | 40 (100%) |  |
| **CD4 cell count [cells/µL]^2^** | n.d. | 449 (28;1004) |  |
| **HIV viral load > 40 copies/mL ^1^** | n.a. | 0 (0%) |  |

**Table S1, Patient characteristics**

1, numbers (percent); 2, median (interquartiles)

n.a., not applicable; n.d. not defined
