# Supplementary material for: Compartment-specific distribution of human intestinal innate lymphoid cells is altered in HIV patients under effective therapy
Source: PLoS Pathog. 2017 May 15;13(5):e1006373. doi: 10.1371/journal.ppat.1006373 (PMC5444854; doi:10.1371/journal.ppat.1006373)
Supplement: S2 Table — (DOCX) [file ppat.1006373.s012.docx]

**Table S2, mAbs used in this study**

| **antigen** | **supplier** | **clone** | **conjugate** | **Panel** |
| --- | --- | --- | --- | --- |
| BDCA2 | Biolegend | AC144 | FITC | 1,2,3 |
| CD1a | Biolegend | HI149 | FITC | 1,2,3 |
| CD3 | Biolegend | UCHT1 | FITC | 1 |
| CD3 | BD | UCHT1 | BUV805 | 2,3,4 |
| CD11c | Biolegend | 3,9 | FITC | 1,2,3 |
| CD14 | Biolegend | HCD14 | FITC | 1,2,3 |
| CD16 | Biolegend | 3G8 | FITC | 1,2,3 |
| CD19 | Biolegend | HIB19 | FITC | 1,2,3 |
| CD34 | Biolegend | 581 | FITC | 1,2,3 |
| CD45 | BD | HI30 | BUV395 | 1,2,3,4 |
| CD56 | Biolegend | HCD56 | BV605 | 1,2,3,4 |
| CD94 | BD | HP-3D9 | FITC | 2,3 |
| CD94 | Novus | 131412 | AF700 | 1,4 |
| CD103 | Novus | Ber-ACT8 | AF700 | 2,3 |
| CD103 | Biolegend | Ber-ACT8 | BV421 | 1,4 |
| CD117 | Biolegend | 104D2 | PE-Cy7 | 1,2,3 |
| CD123 | Biolegend | 6H6 | FITC | 1,2,3 |
| CD127 | Biolegend | A019D5 | APC | 1,4 |
| CD127 | Biolegend | A019D5 | BV711 | 2,3,4 |
| CRTH2 | Biolegend | BM16 | PerCP-Cy5.5 | 1,2,3 |
| EOMES | ebioscience | WD1928 | FITC | 4 |
| EOMES | R&D | 644730 | APC | 2 |
| FCερ1 | Biolegend | AER-37 | FITC | 1,2,3 |
| GATA-3 | Biolegend | 16E10A23 | BV421 | 2 |
| IFN-γ | Biolegend | 4S.B3 | BV421 | 3 |
| IL-13 | Biolegend | JES10-5A2 | PE | 3 |
| IL-22 | R&D | 142928 | APC | 3 |
| NKp44 | Biolegend | P44-8 | Biotin | 1,2,3,4 |
| NKp80 | Miltenyi | 4A4.D10 | FITC | 1,2,3 |
| NKp80 | Biolegend | 5D12 | PE | 4 |
| Rorγt | ebioscience | AFKJS-9 | PE | 2 |
| Streptavidin | Biolegend | - | BV785 | 1,2,3,4 |
| TBET | Biolegend | 4B10 | BV711 | 1,2,3,4 |
| TCRαβ | Biolegend | P26 | FITC | 1,2,3 |
| TCRγδ | Biolegend | B1 | FITC | 1,2,3 |

**grey marking:** antibodies of **lineage cocktail**
