# Supplementary material for: Compartment-specific distribution of human intestinal innate lymphoid cells is altered in HIV patients under effective therapy
Source: PLoS Pathog. 2017 May 15;13(5):e1006373. doi: 10.1371/journal.ppat.1006373 (PMC5444854; doi:10.1371/journal.ppat.1006373)
Supplement: S3 Table — (DOCX) [file ppat.1006373.s013.docx]

**Table S3, Primer sequences**

| **transcript** | **primer sequence** |
| --- | --- |
| forward CXCL-16 | TCT CAA AGA ATG TGG ACA TGC |
| reverse CXCL-16 | CAG GGG TGT GGA TAT CTG AA |
| forward IL-1b | AAG CCC TTG CTG TAG TGG TG |
| reverse IL-1b | GAA GCT GAT GGC CCT AAA CA |
| forward IL-7 | TCC CCT GAT CCT TGT TCT GTT G |
| reverse IL-7 | CGA TGC TGA CCA TTA GAA CAC TC |
| forward IL-15 | CCA TCC AGT GCT ACT TGT GTT TAC TT |
| reverse IL-15 | CCA GTT GGC TTC TGT TTT AGG AA |
| forward IL-18 | ACT GGT TCA GCA GCC ATC TT |
| reverse IL-18 | TGC AGT CTA CAC AGC TTC GG |
| forward IL-22 | TGA ATA ACT AAC CCC CTT TCC CTG |
| reverse IL-22 | TGG CTT CCC ATC TTC CTT TTG |
| forward IL-23a | AGA AGC TCT GCA CAC TGG C |
| reverse IL-23a | CCA CAC TGG ATA TGG GGA AC |
| forward IL-33 | CAA AGA AGT TTG CCC CAT GT |
| reverse IL-33 | AAG GCA AAG CAC TCC ACA GT |
| forward IFN-g | GTA TTG CTT TGC GTT GGA CA |
| reverse IFN-g | TGC TAT TCA AAC TGC CCT GA |
| forward TGF-b1 | TGG CGA TAC CTC AGC AAC C |
| reverse TGF-b1 | CTC GFG GAT CCA CTT CCA G |
| forward TGF-b3 | TAC TAT GCC AAC TTC TGC TCA G |
| reverse TGF-b3 | AAC TTA CCA TCC CTT TCC TC |
| forward TNF-a | GAC CAG GAC TGT GGC TGT |
| reverse TNF-a | ATG AGT GCA GAT GGC ACC |
| forward β-Aktin | CAG GCA CCA GGG CGT GAT GG |
| reverse β-Aktin | CGA TGC CGT GCT CGA TGG GG |
